# Fission–fusion dynamics in the social networks of a North American pitviper

**DOI:** 10.1002/ece3.10339

**Published:** 2023-08-07

**Authors:** Sasha J. Tetzlaff, Jeferson Vizentin‐Bugoni, Jinelle H. Sperry, Mark A. Davis, Rulon W. Clark, Roger A. Repp, Gordon W. Schuett

**Affiliations:** ^1^ U.S. Army ERDC‐CERL Champaign Illinois USA; ^2^ Illinois Natural History Survey, Prairie Research Institute University of Illinois Urbana‐Champaign Champaign Illinois USA; ^3^ Programa de Pós‐Graduação em Biologia Animal, Instituto de Biologia Universidade Federal de Pelotas Pelotas Brazil; ^4^ Department of Natural Resources and Environmental Sciences University of Illinois at Urbana‐Champaign Urbana Illinois USA; ^5^ Chiricahua Desert Museum Rodeo New Mexico USA; ^6^ Department of Biology San Diego State University San Diego California USA; ^7^ NOAO Tucson Arizona USA; ^8^ Department of Biology, Neuroscience Institute Georgia State University Atlanta Georgia USA

**Keywords:** annual migrations, communal living, *Crotalus atrox*, kinship, modularity, non‐random associations, rattlesnake, social environment

## Abstract

Many animal species exist in fission–fusion societies, where the size and composition of conspecific groups change spatially and temporally. To help investigate such phenomena, social network analysis (SNA) has emerged as a powerful conceptual and analytical framework for assessing patterns of interconnectedness and quantifying group‐level interactions. We leveraged behavioral observations via radiotelemetry and genotypic data from a long‐term (>10 years) study on the pitviper *Crotalus atrox* (western diamondback rattlesnake) and used SNA to quantify the first robust demonstration of social network structures for any free‐living snake. Group‐level interactions among adults in this population resulted in structurally modular networks (i.e., distinct clusters of interacting individuals) for fidelis use of communal winter dens (denning network), mating behaviors (pairing network), and offspring production (parentage network). Although the structure of each network was similar, the size and composition of groups varied among them. Specifically, adults associated with moderately sized social groups at winter dens but often engaged in reproductive behaviors—both at and away from dens—with different and fewer partners. Additionally, modules formed by individuals in the pairing network were frequently different from those in the parentage network, likely due to multiple mating, long‐term sperm storage by females, and resultant multiple paternity. Further evidence for fission–fusion dynamics exhibited by this population—interactions were rare when snakes were dispersing to and traversing their spring–summer home ranges (to which individuals show high fidelity), despite ample opportunities to associate with numerous conspecifics that had highly overlapping ranges. Taken together, we show that long‐term datasets incorporating SNA with spatial and genetic information provide robust and unique insights to understanding the social structure of cryptic taxa that are understudied.

## Graphical abstract

Western diamondback rattlesnakes (*Crotalus atrox*) exhibit modular networks centered on reproductive females and stemming from fission/fusion dynamics related to annual cycles and communal denning.

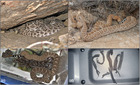

## 1 INTRODUCTION

Since the pioneering works and influential insights of Darwin (1859), Lorenz (1937), Tinbergen (1963), Goodall (2010), and others (Alexander, 1974; Brown, 1975; Dugatkin, 1997; Gowaty, 1996; Hamilton, 1963, 1964; Hinde, 1976; Stamps, 2003; Trivers, 1972), the importance of social behavior, from mate choice, male combat, and territoriality to group hunting, parental care, cooperation, and even play (in humans and other animals), has been inextricably embedded into the foundations of ecological and evolutionary theory (Brown, 1975; Burghardt, 2005; Dugatkin, 2020; Wilson, 1975). Taken to its broadest extent, social behavior in natural populations is defined as *any* interaction that occurs between two or more conspecific or heterospecific individuals (Croft et al., 2008; Doody et al., 2021). Accordingly, social interactions are not only proximate events but also include those occurring at greater distances among individuals.

Historically, social interactions have been analyzed as dyadic interactions, quantified in myriad ways (Allison & Liker, 1982; Bakeman, 1997). The advent of modern network ecology has provided the requisite tools for more nuanced analyses via network theory (Croft et al., 2008; Krause et al., 2015). Although originally developed for studies of human behavior and physics (Croft et al., 2008; Pinter‐Wollman et al., 2014), the conceptual and analytical framework has advanced our understanding of social networks in wild animals (Croft et al., 2008, 2016; Farine, 2017; Farine & Whitehead, 2015; Fortin et al., 2021; Krause et al., 2015; Webber & Vander, 2019). These networks are composed of nodes denoting individuals, groups, or other entities, and edges representing interactions (e.g., behavior) between or among nodes (Appendix A). Importantly, because social network analysis (SNA) provides a conceptual and analytical framework to explore patterns of interconnections among biological entities, it allows researchers to identify emergent group‐level interaction patterns and quantify individuals' contributions to network connectedness. SNA can therefore reveal otherwise unobservable ecological patterns and the processes underlying them (Croft et al., 2008, 2016; Krause et al., 2015). Furthermore, by using null models, robust statistical testing can be achieved to assess whether such emergent group‐level patterns differ from random association among individuals (Farine, 2017; Fortin et al., 2021; Krause et al., 2015).

Social network analysis has elucidated many insights on the social structure of animals, from insects and fishes to primates and cetaceans (Croft et al., 2008, 2016; Krause et al., 2015; Pinter‐Wollman et al., 2014). Yet, despite such advances, there are important gaps, particularly in several specious lineages of vertebrates historically considered to not be highly social (Doody et al., 2021; Schuett, Clark, et al., 2016; Schuett, Repp, et al., 2016). Among these lineages, reptiles in general (but see Godfrey, 2015; Godfrey et al., 2014) and snakes specifically have received particularly short shrift, with scant studies exploring social behavior under the network perspective (Doody et al., 2021). To our knowledge, of the ~4000 extant species, only one snake (*Crotalus cerberus*) has been studied in the wild with these tools (Schuett, Clark, et al., 2016). Most terrestrial snakes have cryptic lifestyles, and structure of their social networks is largely unknown (Doody et al., 2021; Schuett, Repp, et al., 2016). Yet, some species—such as large vipers, boids, and pythonids—are excellent candidates for models of SNA. For example, in many moderate‐ to large‐sized rattlesnakes (e.g., *Crotalus atrox*, *C. cerberus*, *C*. *oreganus*, *C. stephensi*, and *C. viridis*), a variety of factors including large population size and communal winter denning render them desirable subjects to study in nature. Based on prior field research (Amarello, 2012; Clark et al., 2014; Doody et al., 2021; Schuett, Clark, et al., 2016; Schuett, Repp, et al., 2016), it appears most snakes likely form distinct clusters of individuals interacting (i.e., modular networks) which should be defined to some extent by relatedness, but these predictions have yet to be quantitatively tested (Clark et al., 2014; Doody et al., 2021; Schuett, Repp, et al., 2016). Nonetheless, behavioral evidence strongly suggests the existence of fission–fusion dynamics (spatial and temporal changes in the size and composition of conspecific groups) in the social networks of many temperate rattlesnakes (Klauber, 1956; Schuett, Clark, et al., 2016; Schuett, Repp, et al., 2016).

Here, we leveraged long‐term datasets for a population of a large‐bodied North American pitviper, the western diamondback rattlesnake (*Crotalus atrox*), to test hypotheses of social network structure and fission–fusion dynamics (Aureli et al., 2008; Schuett, Clark, et al., 2016; Schuett, Repp, et al., 2016). We asked three main questions: First, do group‐level patterns emerge from distinct social interactions? Second, do individuals' traits influence their connectivity within social networks? Lastly, does genetic relatedness undergird social interactions in this system? We investigated three bipartite interactions (denning, sexual pairing, and parentage) and the drivers of individuals' centrality (Appendix A). Specifically, we tested (a) whether these three bipartite networks presented non‐random modular or nested structure (Appendix A); (b) which attributes (body length, sex, and home range size) are associated with individuals' centrality in these three networks, and (c) whether interactions occurrence and or frequencies in the three social networks and home range overlap are significantly correlated with genetic relatedness among individuals (e.g., kin‐based).

## 2 MATERIALS AND METHODS

### 2.1 Study system

**FIGURE 1:**
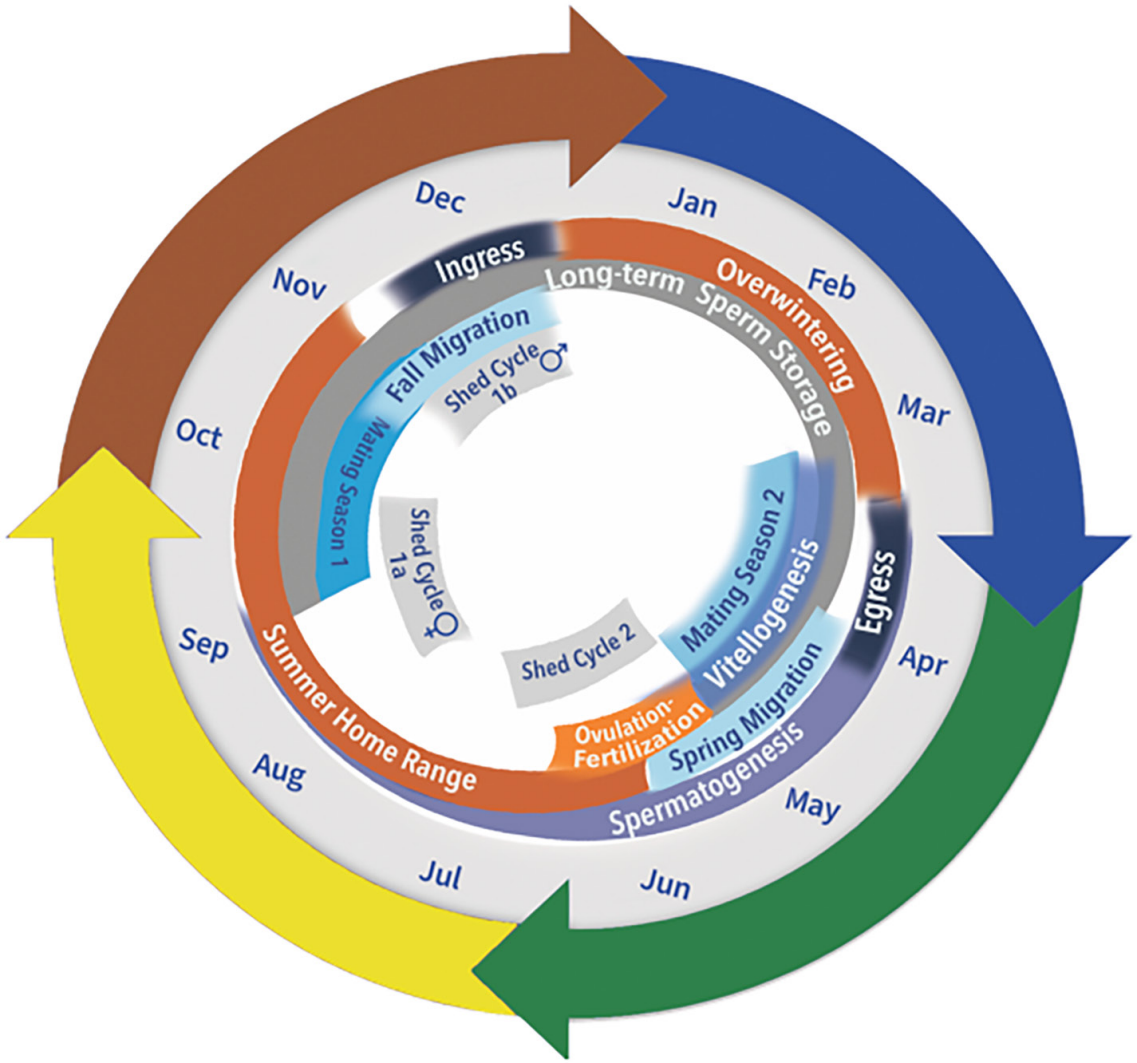
Annual cycle and phenology of behavioral, physiological, reproductive, and life‐history events for adult *Crotalus atrox* at Suizo Mountains (Pinal County, Arizona), and nearby areas, based on 15 consecutive years (2001–2015) of research (Amarello et al., 2010; Greene et al., 2002; Levine et al., 2020, 2021; Repp, 1998; Schuett, Clark, et al., 2016; Schuett, Repp, et al., 2016). Shed cycle refers to skin shedding (ecdysis).

A single population of western diamondback rattlesnakes in the Suizo Mountains (Pinal County, AZ, USA) was studied for 15 consecutive years from March 1, 2001, to December 31, 2015 (Clark et al., 2014; Levine et al., 2020; Schuett, Clark, et al., 2016; Schuett, Repp, et al., 2016). The research site is 40 km SSE of the city of Florence, 8 km W of State Route 79. This region is designated as Sonoran Desert, Arizona Upland Desert‐Scrub subdivision (Clark et al., 2014). Data accumulated for this *C. atrox* population has contributed substantially to our understanding of the species' behavior, reproductive ecology, and life history in Arizona (Repp, 1998; Schuett, Clark, et al., 2016; Schuett, Repp, et al., 2016). Key events of the annual cycle are summarized in Figure 1, but the typical phenology of this population is described for further clarity. Egress from communal dens is centered in late March to early April (Clark et al., 2014; Repp, 1998; Schuett, Clark, et al., 2016; Schuett, Repp, et al., 2016). In most cases, egress lingers—from days to several weeks—and occurs in several phases, including basking at the den entrance (often in groups), making short‐range movements, and returning to the den. The spring mating period (second mating season) occurs before migration movements to their spring home range areas. Courtship and coitus may occur at the den itself or in the general area. Male combat for priority‐of‐access to females also may occur but is rarely observed (Schuett, Clark, et al., 2016; Schuett, Repp, et al., 2016). Migration movements in March and April bring individuals to their spring and summer home ranges. Furthest straight‐line distances traveled from communal dens to home ranges are from several dozen meters to over 2 km (Clark et al., 2014; Schuett, Clark, et al., 2016; Schuett, Repp, et al., 2016); mating (first mating season), skin shedding, and hunting prey are the primary behavioral activities during this time (Clark et al., 2014; Schuett, Clark, et al., 2016; Schuett, Repp, et al., 2016), and except for the two distinct mating seasons, there is generally little contact observed among adults, especially males. In fall (late October through November) adult individuals initiate migration to return to their respective communal dens to re‐establish long‐term social groups (networks) lasting for up to 5 months (November through March). The most common social activity at the communal dens which can be observed in all winter months is termed “sun basking” and occurs at the entrance or alongside the den itself (Repp, 1998; Schuett, Clark, et al., 2016; Schuett, Repp, et al., 2016). Females will sometimes alternate year to year from communal dens to overwintering singly in shelters such as rodent middens and small mammal burrows (Schuett, Clark, et al., 2016; Schuett, Repp, et al., 2016). Males show near absolute fidelity to communal dens but rarely overwinter privately in granitic rubble.

### 2.2 Collecting and processing subjects

Animals selected for this study were either collected at or near known communal dens during egress in spring (March–April) or found in their spring–summer home range. Animals were captured and processed as detailed in previous studies (Amarello et al., 2010; Levine et al., 2020, 2021; Repp, 1998; Schuett, Clark, et al., 2016; Schuett, Repp, et al., 2016). At capture, Global Positioning System (GPS) coordinates were obtained, and subjects were measured (snout–vent length, tail length, head dimensions to the nearest millimeter; body mass to the nearest 1.0 g) and sex confirmed (via probing) while under light anesthesia (isoflurane). Individuals were photographed, implanted with a unique passive integrated transponder (PIT) tag (AVID, Inc.), and their proximal rattle segments were colored via marker. A focal group of adult *C. atrox* collected from 2001 to 2010 were used in social network analyses (*n* = 50 focal animals: 22 males 28 females). Subjects were selected for radio‐tracking based on size (≥700 mm SVL) and good state of health. Each animal had an appropriately sized (≤5% body mass) temperature‐sensitive radio‐transmitter (models SI‐2T and AI‐2T, 11–16 g; Holohil Inc.) surgically implanted within the coelom following general procedures used for snakes (Dormann et al., 2009). After processing, all subjects were released at their exact capture site.

### 2.3 Radio‐tracking

Focal animals were radio‐tracked minimally 2–4 times per month during winter. Tracking was increased substantially—sometimes daily or twice daily—from early August through mid‐September, the period of birthing. During spring and fall, snakes were tracked weekly on average. For each animal location, UTM coordinates were recorded along with behavioral data (particularly if associating with conspecifics), body and environment temperatures, feeding and ecdysis status, plant associations, subject location (above or below the ground surface), visible or not visible, and health status (Amarello et al., 2010; Levine et al., 2020, 2021; Repp, 1998; Schuett, Clark, et al., 2016; Schuett, Repp, et al., 2016).

### 2.4 Spatial analyses

We estimated home range sizes by creating 100% minimum convex polygons (MCPs) around the outermost radiotelemetry locations for each snake in ArcGIS Pro 2.6.1. To produce a single value for the degree of overlap for each possible pair combination of telemetered snakes, we calculated the average overlap for the two individuals in each pair as (AB/A + AB/B)/2, where A is the home range size of individual A, B is the home range size of individual B, and AB is the area shared by both A and B. Using this method, we generated a pairwise matrix of average home range overlap values (Clark et al., 2014).

### 2.5 Genotype data

All social network analyses performed in this study that incorporated DNA‐based information was accomplished using previously published data (Clark et al., 2014; Schuett, Clark, et al., 2016; Schuett, Repp, et al., 2016). See these studies for all procedures used in DNA sampling, extraction, genotyping, and parentage and relatedness analysis.

### 2.6 Social network analysis

**FIGURE 2:**
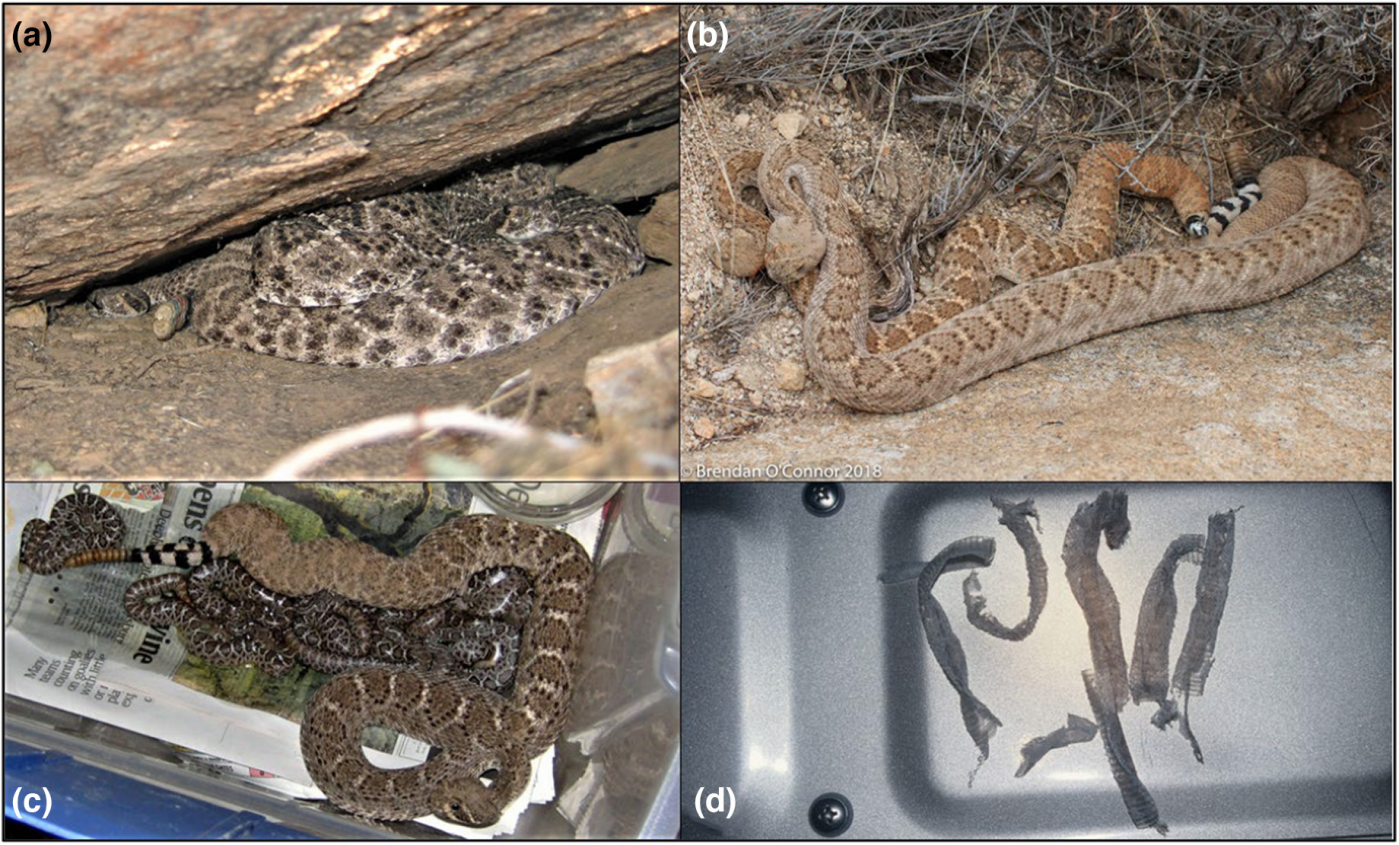
Examples of interactions used to quantify social network structures for western diamondback rattlesnakes (*Crotalus atrox*) inhabiting the Suizo Mountains in Arizona, USA: (a) Adults occupying a communal den, (b) male and female copulating, (c) mother with neonates and (d) shed skins used to genotype individuals. Photographs (a, c, and d) taken by Roger Repp, and B taken by Brendan O'Connor.

We built an interaction matrix for each social interaction considered (denning, pairing, and parentage; Appendix A). The denning network was a matrix of all male and female study subjects as rows and columns containing 1s and 0s indicating whether a given pair of all possible pair combinations of snakes from either sex were observed sharing the same den (Figure 2a) or not, respectively. The pairing network was a matrix with females represented in columns and males represented in rows, containing a series of 1s and 0s indicating whether each possible male–female pair combination was observed engaging in any behavior associated with mating or not, respectively; examples of pairing behavior included male–female pairs in copulation (Figure 2b) or whose bodies were in contact (e.g., males lying on females) or proximity during either mating season. The parentage network was a matrix with females represented in columns and males represented in rows, containing a series of 1s and 0s indicating whether each possible male–female pair combination produced offspring or not, respectively; relatedness among individuals was determined from tissues such as blood or shed skins from adults and neonates (Figure 2c,d).

We tested whether the distribution of interactions among individuals presented modular or nested structure. Modularity was calculated using the metric *Q* and the algorithm DIRTLPAwb+ which searches for the optimum division of the observed interaction matrix into modules (Beckett, 2016). *Q* ranges from 0 to 1 (perfectly modular). Nestedness was estimated using the NODF metric which calculates the non‐overlap and decreasing fill of the interaction matrix (Almeida‐Neto et al., 2008) or WNODF, which is the equivalent for quantitative matrices, that is, interaction frequencies measured (Almeida‐Neto & Ulrich, 2011). Both NODF and WNODF ranges from 0 (no nestedness) to 100 (perfectly nested). We used a null model to test the significance of the observed *Q* and NODF/WNODF by comparing the metric calculated for the observed interaction matrix with the metric calculated from random matrices generated with a null model. Specifically, we used the *vaznull* null model which reshuffles interaction within a matrix while keeping the number of individuals and connectance (i.e., the proportion of realized links in relation to the total possible) the same as the observed matrix. We considered the metric significant when the observed value was higher than the 95% confidence interval produced by the null model. To quantify individuals' connectivity within each network, we calculated their closeness centrality (CC; Appendix A). This metric quantifies the proximity of a node (individual, in this case) to all other nodes in the network (Freeman, 1979; Martín González et al., 2010) indicating nodes that are more connected and, therefore, are highly influential in the network. All analyses were conducted using the *bipartite* package (Dormann et al., 2009) in R version 4.0.5 (R Core Team, 2022).

To investigate whether individual‐specific variables were associated with individual's centrality in a network, we used generalized linear mixed models (GLMMs) where CC was the response variable and sex, snout–vent length (SVL), body mass, and MCP were predictors. Since we detected high correlation between SVL and body mass (Pearson's *r* = .87), we excluded body mass from our models, given lower variation in SVL measurements among individuals (Fortin et al., 2021; Pinter‐Wollman et al., 2014). For snakes tracked more than 1 year, we used mean MCP size as a predictor of CC; adults in this population have highly repeatable annual MCP sizes (*R* = .83, 95% CI: 0.69–0.90; S. J. Tetzlaff et al., unpublished data). For each of the three separate networks, CC was calculated independently and used as the response variable in the models. In all models, we included the number of years an individual was detected as a random factor to control for sampling variation among individuals. Our analyses followed the general recommendations by Zuur et al. (2009). All predictor variables were scaled by mean centering and dividing by the standard deviation prior to analysis. Model fitting was performed using the function *glmmadmb* of the R package *glmmADMB* (Skaug et al., 2018) using *zero‐inflated gaussian* distributions. We then used the function *dredge* of the R package *MuMIn* (Barton, 2019) to compare models including all possible combinations of predictor variables, plus an intercept‐only model. We performed model selection based on the Akaike information criterion corrected for small sample sizes (AIC_c_). Finally, when there was more than one model including >95% of the model weight (Burnham & Anderson, 2002), we conducted model averaging considering all these models.

To test whether genetic relatedness among individuals influenced interactions in any of the three networks, we performed three Mantel tests contrasting each of the three observed matrices with the focal‐animal relatedness matrix (as above). Statistical significance (*α* ≤ .05) was accessed through 9999 permutations, using the R package *ade4* (Thioulouse et al., 2018).

## 3 RESULTS

### 3.1 Subjects

From 2001 to 2015, we sampled 299 individuals: 191 were adult individuals and 108 neonates from 30 different litters produced by 18 different females (Clark et al., 2014). There were 15 additional unmarked males identified via genotyping who sired offspring that were considered in the parentage network (Clark et al., 2014). The denning network was composed of 23 adult individuals (all but two in the focal group with radio‐transmitters), summing up 27 combinations of individuals sharing a den. Few snakes were given IDs during processing at communal shelters and used for aspects of this study (such as relation of relatedness to den choice), but were not radio‐tracked (e.g., CA‐99; Table 1). The pairing network was composed of interactions between 19 females and 28 males, including 43 distinct pairs of individuals. The parentage network was composed of 18 females who sired offspring with 27 males, including 27 distinct pairs of partners siring 46 offspring (see Appendix A: Tables A1, A2, A3, A4, A5, A6, A7, A8).

**TABLE 1:** Data on the use of eight communal dens by a subset (11 adult females, 20 adult males) of the focal group (subjects fitted with radio‐transmitters) of adult *Crotalus atrox* studied in the Suizo Mountains, Arizona (2001–2010).

Winter	Winter communal den ID							
	AD1	AD4	AD5	AD6	AD7	AD8	AD9	AD10
2000–2001	1, 2, 3							
2001–2002	1, 3, 4							
2002–2003	1, 5, 6		31	7				
2003–2004	1, 5, 6, 33, 41	13, 16, 32	34	44, 46	47			
2004–2005	1, 5, 6, 33, 41	13, 16, 32	77	44, 46	47, 58			
2005–2006	1	16, 81		46	47, 76	97, 99	55	98
2006–2007	1	16, 55, 81		44, 46	47, 58, 76	97		98
2007–2008		55			47	102		
2008–2009					47, 92			
2009–2010					79, 122	120		

*Note*: The focal group (*N* = 50) consisted of 22 adult males and 28 adult females. Numbers denote abbreviated IDs of the radio‐tracked subjects (CA‐1 is 1, CA‐5 is 5, and so on) and color denotes sex (orange = females, blue = males). AD = den ID. See text, Appendix A and Appendix S1 for additional information.

### 3.2 Spatial analysis

#### 3.2.1 Landscape use

**FIGURE 3:**
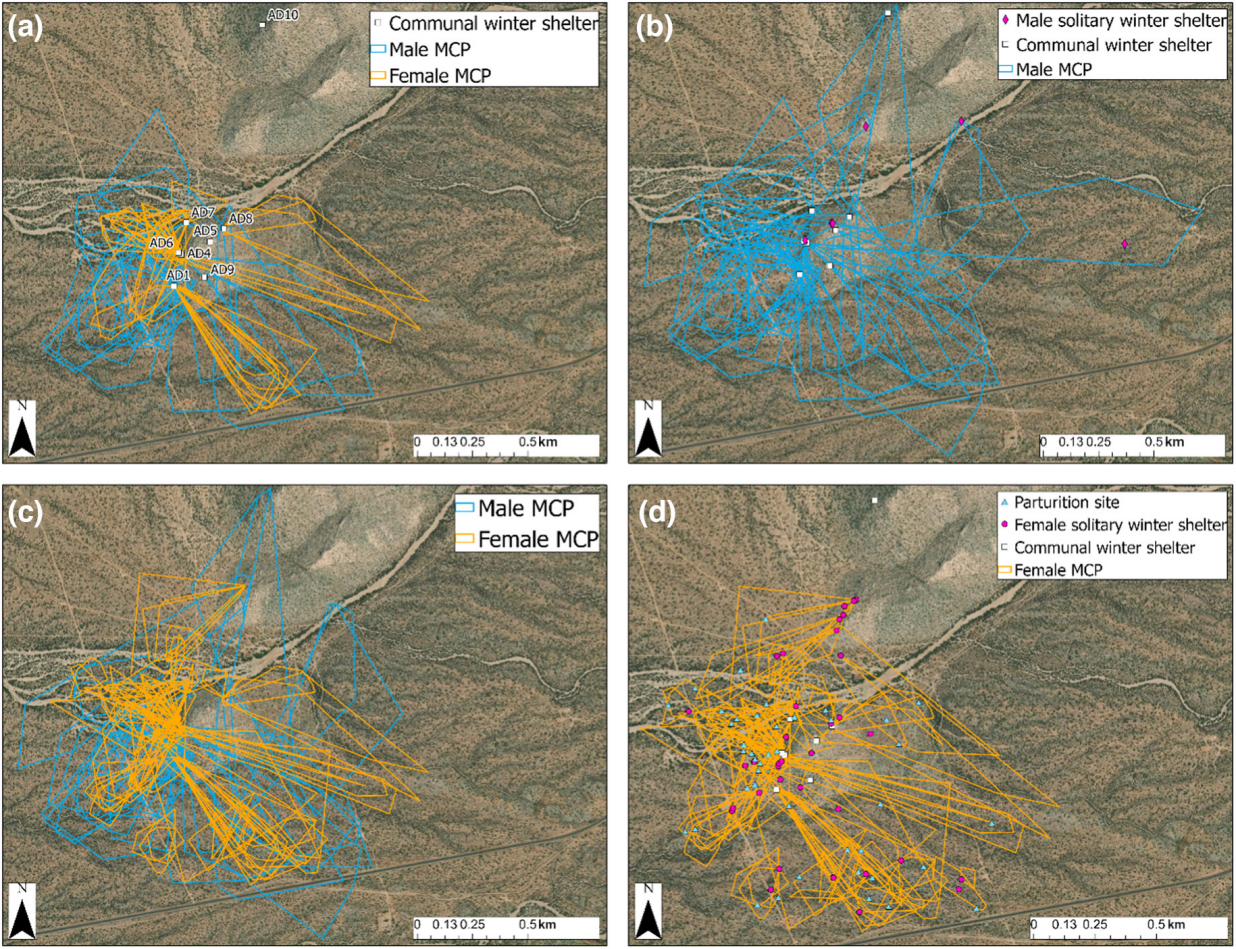
Spatial data on our focal group (22 males, 28 females) of adult *Crotalus atrox*. (a) Annual home ranges (minimum convex polygons; MCP) of males (blue) and females (orange) that were observed overwintering in communal dens (AD = den ID). (b) Sites where males overwintered in isolation (pink diamonds) or communal dens in relation to their annual home range; (c) Home ranges of males and females that were observed engaging in reproductive behaviors. (d) Sites where females gave birth (blue triangles) and overwintered privately (pink circles) or in communal dens (white squares) in relation to their home range.

**FIGURE A1:**
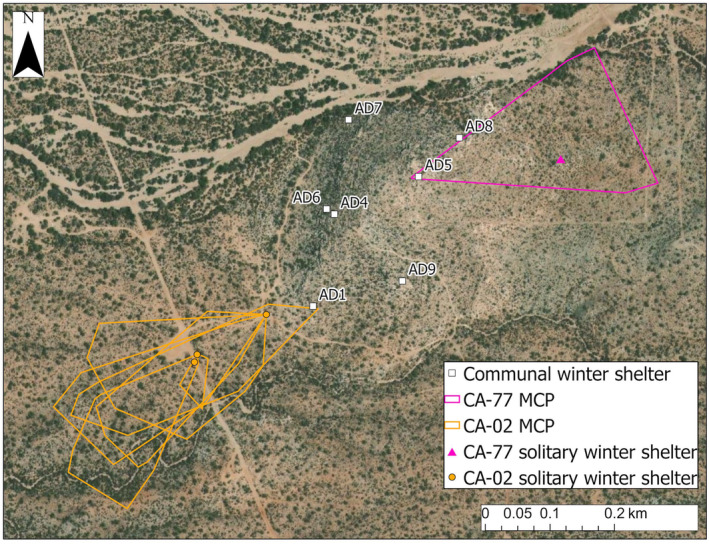
Minimum convex polygons (MCP) showing home ranges for females CA‐02 and CA‐77 that alternated between communal dens (den ID: AD‐1 and AD‐5) and private winter shelters in different years. Note the consistency in CA‐02's annual home range size and shape. See main text for additional details.

Despite considerable individual variation, males had larger and less variable average estimated spring–summer home range sizes (13.36 ha ± 9.26 SD) than females (5.08 ha ± 4.44 SD). MCPs for individuals tracked over multiple years were remarkably consistent in size and shape (see Figure A1 for an example). We observed substantial overlap of many snakes' home ranges, especially for individuals that shared communal dens (Figure 3a) or were observed engaging in reproductive behaviors (Figure 3c). Males largely overwintered in communal dens but would sometimes overwinter in isolation, including at sites far from communal shelters (Figure 3b). Females often gave birth far from communal shelters and overwintered privately much more than males (Figure 3d).

#### 3.2.2 Home range overlap and relatedness

Mantel tests comparing home range (MCP) overlap with relatedness revealed there was no correlation between the degree of pairwise home range overlap and relatedness (*r* = .004, *p* > .05).

### 3.3 Social network analyses

#### 3.3.1 Communal den occupants

With few exceptions, all telemetered individuals that used communal dens exhibited absolute fidelity to these sites over the 10‐year period where snakes were consistently radio‐tracked (Table 1). For example, CA‐1, the longest‐tracked snake in this study, showed fidelity to den AD1 for the seven winters it was tracked. Conversely, females CA‐2 and CA‐77 used a communal shelter for only one winter and were otherwise observed overwintering privately. Similarly, male CA‐55 used a communal shelter twice in three winters (Table 1).

#### 3.3.2 Network structure analysis

**FIGURE 4:**
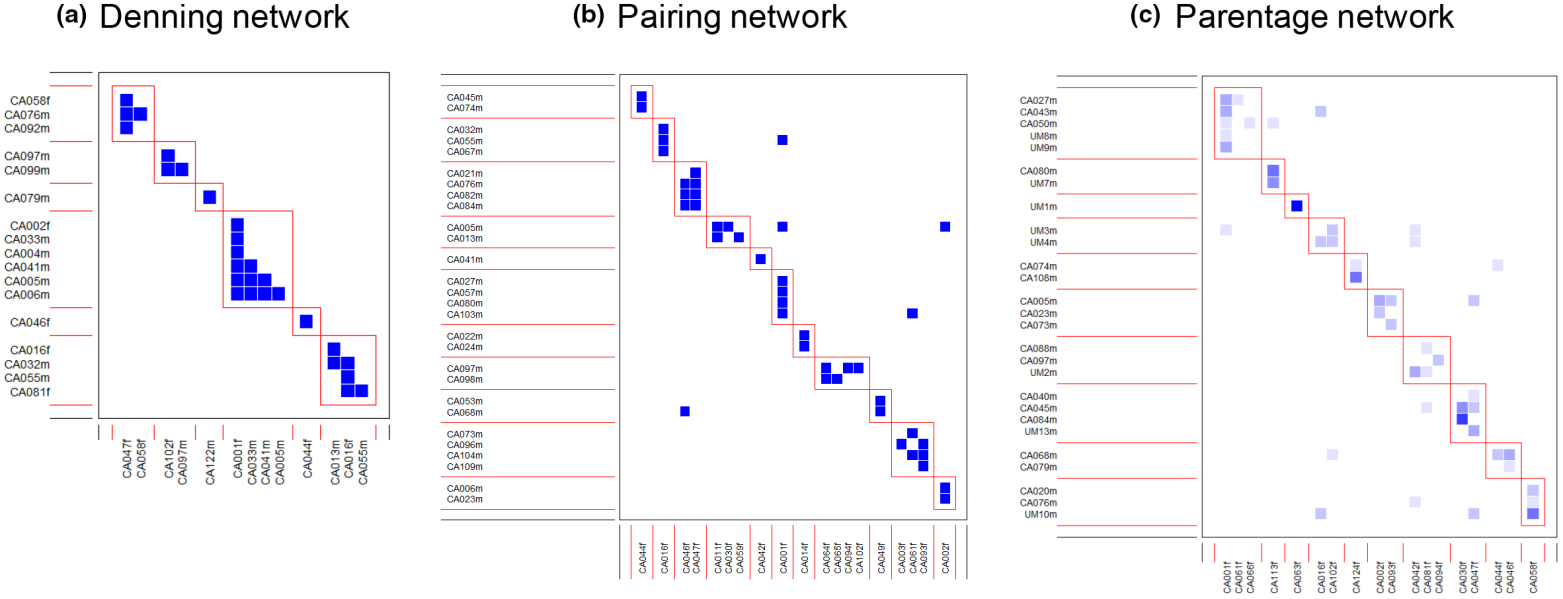
Modularity of individuals' (a) denning network, (b) pairing network, and (c) parentage network of *Crotalus atrox* subjects in this study. Inner boxes indicate subsets of individuals interacting preferentially with each other (i.e., modules). In (a), individuals of both sexes can be at the *x*‐ and *y*‐axes since multiple males and females may share a communal den. In (c) color intensity indicates the number of offspring sired. In (c), UM indicates “unidentified male” (sampled but not radio‐tracked; see text). Nine unidentified males were genotyped in the analysis: UM 1–4, 7–9, and 10, 13. See Dormann et al. (2009). Snakes with IDs that end in “m” are malesand “f” are females.

We detected modularity in all three social networks (denning, pairing, and parentage), but no network was nested. Specifically, the denning network was modular (*Q* = 0.73; 95% CI *Q*_null_ = 0.63–0.72) presenting six modules, including one to six individuals sharing a den, while there was no evidence of nestedness (NODF = 12.80; NODF_null_ = 7.47–15.57). The pairing network was modular (*Q* = 0.77; 95% CI *Q*_null_ = 0.64–0.72) presenting 11 modules including one to four males and one to four females, and there was no evidence of nestedness (NODF = 6.10; NODF_null_ = 5.13–9.23). The parentage network was modular (*Q* = 0.74; 95% CI *Q*_null_ = 0.61–0.73; analysis based on quantitative matrix) presenting 10 modules including one to five males and one to three females and there was no evidence of nestedness (WNODF = 8.21; 95% CI WNODF_null_ = 6.70–12.42). In all three networks, few interactions were recorded outside the modules (*n* = 0 in the denning network, *n* = 5 in the pairing network and *n* = 12 in the parentage network; Figure 4).

Generalized linear mixed models indicated that individuals' CC in the denning, pairing, and parentage networks were not associated with individuals' morphology (SVL) and home range (MCP) (Tables A1, A2, A3, A4, A5, A6). Sex was a significant predictor only in the parentage network, with females having slightly higher CC than males (*β* = −.016, 95% CI: −0.034, −0.002; Table A6).

Overall CC was low, varying from 0 to 0.14 in the denning network, 0 to 0.11 in the pairing network, and 0 to 0.08 in the parentage network (Table A7). Because many individuals were not observed interacting, it was common to have individuals whose CC = 0 (Table A7).

Mantel tests revealed no significant correlations between genetic relatedness and denning (*r* = −.138, *p* = .938), pairing (*r* = .135, *p* = .297), or parentage (*r* = −.150, *p* = .775) networks, which suggests that individuals interacting in modules were unlikely to be closely related.

## 4 DISCUSSION

### 4.1 Overview

In our long‐term study of the behavioral ecology of adult western diamondback rattlesnakes, all three bipartite networks tested were modular and lacked nestedness (Croft et al., 2008; Krause et al., 2015; Newman, 2006). Accordingly, focal animals formed subsets of individuals interacting more frequently with one another than with other individuals (Almeida‐Neto & Ulrich, 2011; Cantor et al., 2017; Dormann et al., 2009). The lack of nestedness indicates that no single individual engaged in interactions with all others, not that less socially connected individuals tend to interact with more socially connected individuals (Almeida‐Neto et al., 2008; Almeida‐Neto & Ulrich, 2011). Our results mirror, to some extent, those of the first and only other study to incorporate social network analysis for a wild snake, the Arizona black rattlesnake, *Crotalus cerberus* (Amarello, 2012; Schuett, Clark, et al., 2016). Adult male and female *C. cerberus* exhibited non‐random association and formed multiple subgroups at communal dens and shelters, yet few dyads had strong associations. More studies are needed to understand the structure of social networks of snakes in general, but we suspect most would not show nested structure. In sharp contrast, the social networks (particularly related to mating) of other terrestrial vertebrates, including African lions (Mbizah et al., 2020), equids and other ungulates (Rubenstein et al., 2015), great apes and other primates (Macdonald & Voelkl, 2015), and several squamates (Bull et al., 2012; Godfrey, 2015; O'Connor & Shine, 2003), are characterized by one or several males that dominate a group of females and likely are the only ones to interact with most or all partners in a group (Krause et al., 2015).

The low CC values obtained reinforces the lack of nestedness and existence of modularity, indicating that each focal subject interacted only with a few other individuals in the three social networks examined. Furthermore, centrality was not significantly predicted by body size, home range, sex, or genetic relatedness. In this social environment, individuals with large bodies or large home ranges do not den, pair with more sexual partners, or produce more offspring than smaller and/or spatially restricted individuals (Dormann et al., 2009; O'Connor & Shine, 2003). Nonetheless, we found females had greater centrality than males in the parentage network—meaning that they produce more offspring with a greater number of partners than males—yet this effect, though statistically significant, is not robust (Levine et al., 2021).

Genetic relatedness of our focal group was not correlated with denning, pairing, or parentage. Spatial analyses revealed that home range overlap also was not significantly correlated with relatedness; thus, social structure between pairs of individuals during the active season was not based on kin associations (Clark et al., 2014; Schuett, Clark, et al., 2016; Appendix S1). Increasingly, studies of other terrestrial vertebrates demonstrate that group living and stable paired associations, for example, are not necessarily kin‐biased or correlated with genetic relatedness (Almeida‐Neto et al., 2008; Baden et al., 2020; but see Godfrey, 2015; Piza‐Roca et al., 2019). In network studies involving lizards, for example, relatedness and group living varies depending on the species (system) being investigated. Group living involves close relatives in some cases (Alexander, 1974; O'Connor & Shine, 2003; Whiting & While, 2017), yet in others, even in strongly connected individuals, social interactions are not defined by relatedness (Godfrey et al., 2014; but see Piza‐Roca et al., 2019). As we discuss later, however, when a larger sample of subjects included unmarked adults (e.g., no radio‐transmitters) was analyzed, the relationship of communal denning and relatedness of *C. atrox* showed mixed results, with multiple communal dens containing related occupants (Schuett, Clark, et al., 2016, pp. 196–198; See Appendix S1, Tables S1–S8).

### 4.2 Social network structure

#### 4.2.1 Annual cycle of communal denning and associated behaviors

The modular and non‐nested structure of the denning network emerged likely via several components of the species' behavior. Communal denning in rattlesnakes has continued to be documented (Klauber, 1956; Schuett, Clark, et al., 2016; Schuett, Repp, et al., 2016; Sexton et al., 1992), most extensively in timber rattlesnakes (*Crotalus horridus*) in the northeastern United States. Adult and juvenile *C. horridus* typically use communal dens (termed hibernacula), exhibiting high levels of natal den philopatry. In the western United States, numerous rattlesnake species are known to use communal dens in winter, but sometimes are active year‐round (Amarello et al., 2010; Brown, 2016; Graves & Duvall, 1995; Repp, 1998; Schuett, Clark, et al., 2016; Schuett, Repp, et al., 2016; Sexton et al., 1992). These species that den communally often show high fidelity to dens (reviewed in Schuett, Clark, et al., 2016). However, studies lasting >5 years on individual occupancy and behavioral activities at communal dens remain rare (Booth & Schuett, 2011; Brown, 2016; Loughran et al., 2015; Schuett, Clark, et al., 2016; Schuett, Repp, et al., 2016).

The social ecology of adult male and female *C. atrox* at the Suizo Mountain site represents fission–fusion dynamics, per Aureli et al. (2008), associated with annual migrations to spring–summer home range areas and fidelity to communal winter shelters (Clark et al., 2014; Repp, 1998; Schuett, Clark, et al., 2016; Schuett, Repp, et al., 2016). In this system, adults occupy specific microhabitats throughout their respective spring–summer home ranges of the Sonoran Desert landscape. During spring and summer, mating, shedding, and hunting prey are the primary behavioral activities (Clark et al., 2014; Schuett, Clark, et al., 2016; Schuett, Repp, et al., 2016), with generally little contact observed among adults (especially males) despite substantial overlap in home ranges. On several occasions, females were found together on the ground's surface, or inside a mammal burrow or midden, and sometimes this is associated with birthing (Schuett, Clark, et al., 2016; Schuett, Repp, et al., 2016). However, birthing rookeries described in other rattlesnakes, which sometimes consist of a dozen or more females (Graves & Duvall, 1995), were never observed in *C. atrox*, nor were communal dens used as birthing sites in this study (Figure 3). Furthermore, neonates and juveniles were never observed at communal dens; thus, we presume they were isolated in rodent middens, small mammal burrows, or similar types of shelters during winter.

#### 4.2.2 Communal denning and relatedness

Based on Hamilton's foundational insights (Hamilton, 1963, 1964), and others' subsequent work (Alexander, 1974; Trivers, 1972; Wilson, 1975), we have a robust understanding of the evolutionary benefits and costs of group living (Dugatkin, 1997; Wittenberger, 1981). Benefits for both kin‐ and non‐kin‐based social groups include increased vigilance to predators and enemies, protection from the environment, increased opportunities for reproduction, and the expression of social behaviors including grooming and parental duties (e.g., uniparental, biparental, and helpers). Living in exclusive kin‐based groups, such as families (Emlen, 1995) offers individuals opportunities for increasing their inclusive fitness, among other benefits (Hamilton, 1964). Under these conditions cooperative and altruistic behaviors can evolve, such as forsaking reproduction and caring for the progeny of relatives (Hamilton, 1963). Such tight kin‐based groups are known for certain groups of lizards (Davis et al., 2011; Doody et al., 2021; Gardner et al., 2016; O'Connor & Shine, 2003), but not in snakes (Doody et al., 2021). Living in groups also has costs, which can be severe, and include the spread of parasites and disease, limited numbers of mates, and competition for food and space itself (Dugatkin, 1997; Evans et al., 2015; Wittenberger, 1981).

Our bipartite analysis of the focal group of adult *C. atrox* showed that relatedness was not a significant component of the social structure of occupants at communal dens. Only 23 of 50 focal subjects were associated with communal dens (Figure 4a). However, using a larger sample of individuals derived from previous analyses (Clark et al., 2014; Schuett, Clark, et al., 2016), overall genetic relatedness among dens was significantly greater than random (Appendix S1, Tables S1–S8). Although kin recognition has not been documented in *C. atrox*, it has been documented in other pitvipers, including rattlesnakes (Clark, 2004; Hoss et al., 2015). Thus, we suspect that *C. atrox* shares this capacity for kin recognition, even if it does not appear to be a major driver of its social networks. Empirical studies could determine whether kin recognition is operating at the communal dens and expressed in social preferences (Clark et al., 2012). In the only other study of rattlesnakes where relatedness of occupants of communal dens has been measured, juveniles and pregnant females preferentially associate with kin under certain conditions, yet communal denning was not kin‐based (Clark et al., 2012).

#### 4.2.3 Social groups versus aggregations

Communal denning is a type of clumped spacing behavior often defined as “aggregation” (Schuett, Clark, et al., 2016). However, with respect to *C. atrox* in this study, and likely other rattlesnake species, we abandon use of the term “aggregation” and alter the lexicon by defining communal denning as *the formation of social groups or colonies by individual preference*. We suggest that these groups form and evolve through mutual attraction of individuals (regardless of members' relatedness) for cooperative benefits to survival and reproduction (Evans et al., 2015; Hamilton, 1964; Hatchwell, 2010). These social groups we observed, whether kin‐ or non‐kin‐based, occur seasonally in a predictable manner. Importantly, these social groups involve only a subset of adult individuals, occurring in microhabitats that are not limited in the local population. These traits indicate that social groups are not just a result of attraction to particular microhabitats. It is likely that communal denning behavior, such as in *C. atrox* and other snakes, may be coordinated by way of conspecific attraction or familiarity, resulting in social (communication) networks which ultimately leads to the partitioning of individuals into subgroups and to the observed network modularity (Croft et al., 2008; Hatchwell, 2010; Krause et al., 2015).

Remarkably, over the 15‐year period of study, the focal group of adults showed near‐absolute fidelity to communal den sites. Several females, however, alternated year to year from communal dens to overwintering singly in shelters such as rodent middens and small mammal burrows (Schuett, Clark, et al., 2016; Schuett, Repp, et al., 2016). Adult males in our population, on the other hand, never occupied these kinds of temporary structures during the cooler months (November through March). This sexually dimorphic behavior related to den use in winter has not been described, to our knowledge, for any snake species (Amarello et al., 2010; Repp, 1998; Sexton et al., 1992). Furthermore, because we never observed neonates or juveniles of *C. atrox* at the communal dens used by the adults; we presume that they were isolated and alone in rodent middens or small mammal burrows during winter. This also contributes to the high level of modularity observed of the denning network.

#### 4.2.4 Pairing network

Emerging research on the social environment increasingly reveals that sexual selection is dynamic, varying both temporally and spatially. Moreover, individuals frequently select for specific social environments, with direct implications on fitness (Bull et al., 2012; McGlothlin et al., 2010; Wolf et al., 1999) as social conditions (e.g., population density, opportunities for interaction, etc.) provide critical contexts for sexual selection (Oh & Badyaev, 2010; Procter et al., 2012). However, this can be buttressed by relatedness as related individuals may be less likely to harm conspecifics (Pizzari et al., 2015) or more likely to disperse to avoid harm (Faria et al., 2019), ostensibly improving individuals' inclusive fitness.

Although some interactions related to mating behavior were likely not observed because snakes were intermittently located with radiotelemetry, in our study we revealed that the pairing network is modular and largely driven by focal females interacting via reproduction‐linked behaviors with multiple males, and often during fusion events at winter shelters. Perhaps more interestingly, some female *C. atrox* in this network occasionally leave communal dens they historically occupied, overwinter solitarily, and then return to their preferred communal dens in subsequent years. The mechanism behind this phenomenon is unclear, but it suggests that females are modulating their participation in the social environment, perhaps with considerable fitness consequences. We speculate that because pitvipers are generally capital breeders, conditions might arise in which lack of resources would render reproducing risky in the following season. Thus, by modulating their social environment, females may exert some control over reproductive output. And while we did not find a positive association between pairing and relatedness among focal (i.e., radio‐telemetered) animals, positive relatedness among all individuals in communal dens was revealed in previous studies of this system (Schuett, Clark, et al., 2016; Schuett, Repp, et al., 2016).

#### 4.2.5 Parentage network

Recent studies show that the social environment itself may influence the pattern of paternity levels in general, and multiple paternity among individuals specifically (Baden et al., 2020; Bull et al., 2012). Furthermore, the social environment can effectively modulate the degree of multiple paternity based on the structure of the social network itself (Bull et al., 2012; Cohas & Allainé, 2009; Dugdale et al., 2007; Martín González et al., 2010). Concomitant with the results of the pairing network, we recover a similar modular pattern that features several focal females, each one producing offspring with a subset of males, and often exhibiting multiple paternity. This is reflective of both the social environment and the biology of *C. atrox*. First, the two distinctly different annual mating periods present decidedly different reproductive contexts. The first mating period occurs late in the active season and out on the landscape, where snakes in this population are seldom observed interacting and are less likely to encounter large numbers of conspecifics. Conversely, the second mating period occurs at or near communal dens shortly after spring egress. In this context, the opportunity for multiple matings increases, ostensibly elevating the probability of multiple paternity. In addition, despite data that indicate communal denning (social groups) in this population show some level of relatedness (Schuett, Clark, et al., 2016; Schuett, Repp, et al., 2016), the parentage network of our focal animals was not positively correlated with relatedness, which is indicative of some degree of either assortative mating or inbreeding avoidance. Ultimately, the social environment coupled with the species biology appears to promote elevated levels of multiple paternity but depress the degree of inbreeding among males and those females acting as nodes in the social network.

## 5 CONCLUSIONS

Nearly three decades ago the first theoretic analysis of snake mating systems was proposed (Duvall et al., 1993) which identified, characterized, and quantified snake mating systems within formal selection theory (Arnold & Duvall, 1994). Over the years, a wealth of new information on population genetics, behavior, reproduction, sexual selection, and parental care of snakes has emerged (Booth & Schuett, 2011, 2016; Greene et al., 2002; Jellen & Aldridge, 2011; Levine et al., 2021; Rivas & Burghardt, 2005; Schuett, Clark, et al., 2016; Schuett, Repp, et al., 2016). We contend that the incorporation of social network analysis into studies documenting spatial ecology, habitat use, and genetic relatedness represents another major methodological advance that can provide novel insights and directions for future research (Schuett, Clark, et al., 2016; Schuett, Repp, et al., 2016). Specifically, we demonstrated strong fission–fusion dynamics, particularly with respect to annual migrations to spring–summer home ranges and use of communal dens during winter by a combination of social preference and experience; relatedness (kin association) also may play a role. Although this fission–fusion behavior has not been formally reported for other snake species, we suggest it may be more common than currently recognized and urge researchers to leverage myriad existing similar datasets to further quantify social network structures for such cryptic and understudied species. Arguably, our study advances the understanding of individuals' reproductive strategies within populations and between the sexes by identifying determinants of social interaction patterns and individual fitness and lays the foundation for additional research into the social ecosystem of cryptic taxa.

Despite this substantial progress in our understanding of snake mating systems, several important issues are problematic and remain unresolved. We conclude with four intriguing examples. Our view is that social networking analyses similar to those we have employed here will be a critical method necessary for addressing all of these questions.

First, perhaps the most perplexing issue in our system is the presence of two distinct mating seasons, which is rarely present in other reptiles (Clark et al., 2014; Graham et al., 2008; Schuett, Clark, et al., 2016; Schuett, Repp, et al., 2016). To date, there has been little theoretical research into this phenomenon. The two annual reproductive periods are temporally distinct and present decidedly different socio‐ecological contexts. Furthermore, because these distinct mating periods occur prior to ovulation in late spring (Schuett, Clark, et al., 2016; Schuett, Repp, et al., 2016; Figure 1), increased mating opportunities for both sexes provide a possible adaptive explanation for their occurrence (Arnold & Duvall, 1994; Duvall et al., 1993; Parker & Birkhead, 2013; Shuster et al., 2013). Whether or not *C. atrox* has two distinct mating seasons across its expansive geographical range in the United States and Mexico is unknown.

Second, the adaptive significance of polyandry in *C. atrox*, as in other animals, is difficult to reconcile. Female *C. atrox* (and females in many other viperids) can have several different mating partners per annum. Why should females mate with multiple partners for fertilization of a single litter, especially in cases where female fecundity does not increase with multiple mating (Duvall et al., 1993), as in this system (Clark et al., 2014; Schuett, Clark, et al., 2016; Schuett, Repp, et al., 2016)? Numerous adaptive explanations have been proposed but have yet to be investigated empirically. For example, by having several partners per annum the likelihood of multiple paternity increases and thus the possibility for greater genetic and phenotypic diversity per litter (including, perhaps, diversity of social behaviors) (Clark et al., 2014; Parker & Birkhead, 2013; Shuster et al., 2013), which could result in higher overall lifetime reproductive success despite the inherent costs incurred by mating multiply (e.g., increased energetic investment, exposure to disease, and predation).

Third, information on how individual *C. atrox* and other snakes first come to learn and delineate their home range and other preferred spatial locations (such as communal dens) is sparse, at best. For instance, is a snake's home range inherited from, or part of, their mother's home range, and thus is acquired and learned via maternal social transmission (Hobaiter et al., 2014; Ilany & Akcay, 2016; Szabo et al., 2021)? We envision this process might be further facilitated by behavioral (kin‐ and self‐recognition, individuality) and chemosensory (pheromones) social information gleaned from conspecifics (Burghardt et al., 2021; Cobb et al., 2005; Ford, 1986; Mason & Parker, 2010; Muellman et al., 2018; Waters et al., 2017). As technological advances debut, particularly the miniaturization of tracking devices (Beaupre, 2016), understanding the ontogeny of home range development, social networks, and space use in snakes from birth to adulthood will be important to explore (Schuett, Clark, et al., 2016; Schuett, Repp, et al., 2016).

Finally, we are aware of populations of *C. atrox*, even within several kilometers of our study site at the Suizo Mountains that do not exhibit communal denning (Schuett, Clark, et al., 2016; Schuett, Repp, et al., 2016). To a large extent, the type of landscape (e.g., geology) appears to dictate whether communal denning is present in a population, and thus could be a critical additional data layer to incorporate into social network analyses. For example, we have noted that igneous rock formations are not used by *C. atrox* for communal denning (Schuett, Clark, et al., 2016; Schuett, Repp, et al., 2016). Though vastly understudied, landscape configuration is showing to be a potent driver of and context for the social environment of animals, thereby shaping the ecology and evolution of societies and their cultures including those of snakes and other reptiles (He et al., 2019).

## AUTHOR CONTRIBUTIONS

**Sasha J. Tetzlaff:** Conceptualization (equal); formal analysis (equal); investigation (equal); methodology (equal); writing – original draft (equal); writing – review and editing (equal). **Jeferson Vizentin‐Bugoni:** Conceptualization (equal); data curation (equal); formal analysis (equal); investigation (equal); visualization (equal); writing – original draft (equal); writing – review and editing (equal). **Jinelle H. Sperry:** Conceptualization (equal); investigation (equal); writing – review and editing (equal). **Mark A. Davis:** Conceptualization (equal); data curation (equal); formal analysis (equal); investigation (equal); methodology (equal); project administration (equal); supervision (equal); visualization (equal); writing – original draft (equal); writing – review and editing (equal). **Rulon W. Clark:** Conceptualization (equal); data curation (equal); formal analysis (equal); investigation (equal); writing – review and editing (equal). **Roger A. Repp:** Conceptualization (equal); data curation (equal); investigation (equal); writing – review and editing (equal). **Gordon W. Schuett:** Conceptualization (equal); data curation (equal); formal analysis (equal); funding acquisition (equal); investigation (equal); methodology (equal); project administration (equal); supervision (equal); validation (equal); writing – original draft (equal); writing – review and editing (equal).

## FUNDING INFORMATION

This study was funded by a Research Incentive Award (US National Science Foundation) and a Research Creative Activities Award (Arizona State University West) to GS. Arizona State University, Zoo Atlanta, Georgia State University, San Diego State University, The University of Tulsa, and David L. Hardy Sr contributed funding support.

## CONFLICT OF INTEREST STATEMENT

The authors declare no conflicts of interest.

## Supporting information


Appendix S1

## Data Availability

The data used in the present analysis are available from the Dryad Digital Repository: https://doi.org/10.5061/dryad.3xsj3txjr.

## APPENDIX A

### Definitions of important terms in social network ecology that were used in the present study

#### Association index

Any measure of the strength of association between two species (Croft et al., 2016; Godfrey et al., 2014).

#### Centrality

The extent to which a given node (e.g., individual) occupies a position that is important in the structure of the network (Croft et al., 2016; Godfrey et al., 2014).

#### Closeness centrality

A measure of centrality that quantifies the proximity of a node (e.g., individual) to all other nodes in the network and thus indicates nodes that are more connected and highly influential in the social network (Graves & Duvall, 1995; Loughran et al., 2015).

#### Edge

A line between two nodes (e.g., individuals) representing a social interaction (Croft et al., 2016; Godfrey et al., 2014).

#### Fission–fusion dynamics

The extent of variation in spatial cohesion and individual membership in a group over time (R Core Team, 2022).

#### Modularity

A measure of subsets (groups, clusters, or communities) of entities (e.g., individuals) that interact with each other more frequently than with other individuals in a population; groups or modules of highly connected individuals. High modularity networks have dense connections between nodes within modules but few connections (between nodes) in different modules (Baden et al., 2020; Godfrey et al., 2014).

#### Nestedness

Interactions of less connected elements (e.g., individuals) that form proper subsets of the interactions of more connected elements, for example, individuals (Godfrey et al., 2014; Hoss et al., 2015; Piza‐Roca et al., 2019; Whiting & While, 2017).

#### Node

An object in a network, such as an individual (Croft et al., 2016; Godfrey et al., 2014).

#### Social preference

Selection of one element (e.g., individual) more frequently over another element (e.g., individual) in the context of a social environment. Non‐random, repeated interactions with certain individuals that are the foundation of social relationships. Also termed preferred association (Bakeman, 1997; Croft et al., 2016; Godfrey et al., 2014; Krause et al., 2015).

### Network analysis details

In an interaction matrix, each node (column *i* or row *j*) represents an individual and each social interaction observed between two individuals (*a*_*ij*_) is an edge. Modularity occurs when subsets of individuals interact more among themselves than with other individuals in the population, forming modules of highly connected individuals.

Nestedness occurs when highly connected individuals interact with each other, while the less connected individuals only interact with a subset of the partners of the most connected individuals.

$${\mathrm{CC}}_{i}=\sum_{j=1;i\neq j}^{n}\frac{d_{\mathrm{ij}}}{n-1}$$

where *n* is the number of individuals in the network and *d*_*ij*_ is the shortest distance between individuals *i* and *j*. CC_*i*_ ranges from 0 to 1 with values closer to 1 indicating higher connectivity of an individual in relation to all others in the population (Loughran et al., 2015).

**TABLE A1:** Model selection results (encompassing 95% of the total model weight) for predicting the effects of snout–vent length (SVL), home range size (MCP), and sex on closeness centrality (CC) for western diamondback rattlesnakes (*Crotalus atrox*) in a network based on observations of adult pairs overwintering in the same den shelter.

Model	df	logLik	AIC_c_	∆AIC_c_	Weight
Sex	5	89.11	−166.82	0	0.31
Sex + MCP	6	90.24	−166.47	0.35	0.26
(Intercept)	4	87.43	−165.95	0.88	0.20
Sex + SVL	6	89.16	−164.33	2.50	0.09
SVL	5	87.73	−164.06	2.77	0.08
Sex + MCP + SVL	7	90.25	−163.77	3.05	0.07

*Note*: Referred to as the “denning network” in main text. ΔAIC_c_ is the difference in AIC_c_ values from a given model to the top‐ranked model. AIC_c_ weight shows the relative likelihood a given model is the most supported. + indicates an additive effect.

**TABLE A2:** Model averaged parameter estimates (*β*) and estimates of their precision for predicting closeness centrality (CC) for adult western diamondback rattlesnakes (*Crotalus atrox*) in a network based on observations of adult pairs overwintering in the same den shelter.

Predictor	*β*	Adjusted SE	Lower 95% CI	Upper 95% CI
(Intercept)	.018	0.013	−0.007	0.043
Sex	.026	0.014	−0.001	0.054
MCP	−.010	0.007	−0.022	0.003
SVL	.001	0.007	−0.014	0.014

*Note*: Referred to as the “denning network” in main text. The 95% confidence interval (CI) includes zero for all predictors.

Abbreviations: MCP, home range size minimum convex polygon; SVL, snout–vent length.

**TABLE A3:** Model selection results (encompassing 95% of the total model weight) for predicting the effects of snout–vent length (SVL), home range size (minimum convex polygon, MCP), and sex on closeness centrality (CC) for western diamondback rattlesnakes (*Crotalus atrox*) in a network based on observations of adult pairs engaging in behaviors related to reproduction.

Model	df	logLik	AIC_c_	∆AIC_c_	Weight
SVL	5	107.22	−203.05	0	0.63
SVL + MCP	6	107.72	−201.43	1.62	0.28
MCP + Sex	6	106.54	−199.08	3.97	0.09

*Note*: Referred to as “pairing network” in the main text. ΔAIC_c_ is the difference in AIC_c_ values from a given model to the top‐ranked model. AIC_c_ weight shows the relative likelihood a given model is the most supported. + indicates an additive effect.

Abbreviations: MCP, home range size minimum convex polygon; SVL, snout–vent length.

**TABLE A4:** Model averaged parameter estimates (*β*) and estimates of their precision for predicting closeness centrality (CC) for adult western diamondback rattlesnakes (*Crotalus atrox*) in a network based on observations of adult pairs engaging in behaviors related to reproduction.

Predictor	*β*	Adjusted SE	Lower 95% CI	Upper 95% CI
(Intercept)	.037	0.012	0.014	0.060
SVL	−.008	0.006	−0.019	0.005
MCP	.004	0.004	−0.005	0.011
Sex	−.004	0.009	−0.022	0.014

*Note*: Referred to as “pairing network” in the main text. The 95% confidence interval (CI) includes zero for all predictors.

Abbreviations: MCP, home range minimum convex polygon; SVL, snout–vent length.

**TABLE A5:** Model selection results (encompassing 95% of the total model weight) for predicting the effects of sex and home range size (MCP) on closeness centrality (CC) for a network describing the number of offspring produced between pairs of western diamondback rattlesnakes (*Crotalus atrox*).

Model	df	logLik	AIC_c_	∆AIC_c_	Weight
Sex	5	109.15	−206.90	0	0.61
Sex + MCP	6	109.29	−204.58	2.31	0.20
MCP	5	107.76	−204.12	2.77	0.18

*Note*: Referred to as “parentage network” in the main text. ΔAIC_c_ is the difference in AIC_c_ values from a given model to the top‐ranked model. AIC_c_ weight shows the relative likelihood a given model is the most supported. + indicates an additive effect. SVL is not presented as it did not contribute models with weight.

Abbreviations: MCP, home range size minimum convex polygon; SVL, snout–vent length.

**TABLE A6:** Model averaged parameter estimates (*β*) and estimates of their precision for predicting closeness centrality (CC) for a network describing the number of offspring produced between pairs of western diamondback rattlesnakes (*Crotalus atrox*).

Predictor	*β*	Adjusted SE	Lower 95% CI	Upper 95% CI
(Intercept)	.037	0.008	0.020	0.054
MCP	−.002	0.004	−0.013	0.005
**Sex (male)**	**−.016**	**0.010**	**−0.034**	**−0.002**

*Note*: Referred to as “parentage network” in the main text. Bold indicates the 95% confidence interval (CI) did not cross zero for sex.

Abbreviation: MCP, home range minimum convex polygon.

**TABLE A7:** Closeness centrality (CC) for each male and female *Crotalus atrox* on the pairing, parentage, and denning networks.

Pairing network			Parentage network			Denning network		
ID	Sex	CC	ID	Sex	CC	ID	Sex	CC
CA001f	Female	0.11196229	CA001f	Female	0.08057908	CA001f	Female	0.14285714
CA002f	Female	0.08986447	CA002f	Female	0.05285441	CA002f	Female	0.00000000
CA003f	Female	0.05362404	CA003f	Female	0.00000000	CA014f	Female	0.00000000
CA008f	Female	0.00000000	CA008f	Female	0.00000000	CA016f	Female	0.09523810
CA010f	Female	0.00000000	CA010f	Female	0.00000000	CA029f	Female	0.00000000
CA011f	Female	0.09870359	CA011f	Female	0.00000000	CA030f	Female	0.00000000
CA012f	Female	0.00000000	CA012f	Female	0.00000000	CA039f	Female	0.00000000
CA014f	Female	0.00000000	CA014f	Female	0.00000000	CA042f	Female	0.00000000
CA015f	Female	0.00000000	CA015f	Female	0.00000000	CA044f	Female	0.00000000
CA016f	Female	0.06923984	CA016f	Female	0.08057908	CA046f	Female	0.00000000
CA017f	Female	0.00000000	CA017f	Female	0.00000000	CA047f	Female	0.04761905
CA019f	Female	0.00000000	CA019f	Female	0.00000000	CA049f	Female	0.00000000
CA029f	Female	0.00000000	CA029f	Female	0.00000000	CA058f	Female	0.04761905
CA030f	Female	0.08986447	CA030f	Female	0.05422016	CA059f	Female	0.00000000
CA039f	Female	0.00000000	CA039f	Female	0.00000000	CA061f	Female	0.00000000
CA042f	Female	0.00000000	CA042f	Female	0.07784758	CA062f	Female	0.00000000
CA044f	Female	0.00000000	CA044f	Female	0.05941000	CA064f	Female	0.00000000
CA046f	Female	0.03535651	CA046f	Female	0.05531276	CA066f	Female	0.00000000
CA047f	Female	0.02651738	CA047f	Female	0.07716471	CA077f	Female	0.00000000
CA049f	Female	0.02651738	CA049f	Female	0.00000000	CA081f	Female	0.00000000
CA051f	Female	0.00000000	CA051f	Female	0.00000000	CA093f	Female	0.00000000
CA056f	Female	0.00000000	CA056f	Female	0.00000000	CA094f	Female	0.00000000
CA058f	Female	0.00000000	CA058f	Female	0.06487299	CA095f	Female	0.00000000
CA059f	Female	0.06393636	CA059f	Female	0.00000000	CA100f	Female	0.00000000
CA060f	Female	0.00000000	CA060f	Female	0.00000000	CA102f	Female	0.04761905
CA061f	Female	0.08544490	CA061f	Female	0.05053264	CA120f	Female	0.00000000
CA062f	Female	0.00000000	CA062f	Female	0.00000000	CA121f	Female	0.00000000
CA063f	Female	0.00000000	CA063f	Female	0.00000000	CA124f	Female	0.00000000
CA064f	Female	0.05303477	CA064f	Female	0.00000000	CA004m	Male	0.00000000
CA065f	Female	0.03535651	CA065f	Female	0.00000000	CA005m	Male	0.14285714
CA066f	Female	0.00000000	CA066f	Female	0.05462988	CA007m	Male	0.00000000
CA070f	Female	0.00000000	CA070f	Female	0.00000000	CA013m	Male	0.07142857
CA071f	Female	0.00000000	CA071f	Female	0.00000000	CA031m	Male	0.00000000
CA077f	Female	0.00000000	CA077f	Female	0.00000000	CA032m	Male	0.00000000
CA081f	Female	0.00000000	CA081f	Female	0.06487299	CA033m	Male	0.14285714
CA085f	Female	0.00000000	CA085f	Female	0.00000000	CA034m	Male	0.00000000
CA086f	Female	0.00000000	CA086f	Female	0.00000000	CA037m	Male	0.00000000
CA087f	Female	0.00000000	CA087f	Female	0.00000000	CA038m	Male	0.00000000
CA089f	Female	0.00000000	CA089f	Female	0.00000000	CA041m	Male	0.14285714
CA090f	Female	0.00000000	CA090f	Female	0.00000000	CA050m	Male	0.00000000
CA093f	Female	0.07218621	CA093f	Female	0.05285441	CA055m	Male	0.07142857
CA094f	Female	0.04419564	CA094f	Female	0.00000000	CA006m	Male	0.00000000
CA095f	Female	0.00000000	CA095f	Female	0.00000000	CA076m	Male	0.00000000
CA100f	Female	0.00000000	CA100f	Female	0.00000000	CA079m	Male	0.00000000
CA101f	Female	0.00000000	CA101f	Female	0.00000000	CA092m	Male	0.00000000
CA102f	Female	0.04419564	CA102f	Female	0.07784758	CA096m	Male	0.00000000
CA112f	Female	0.00000000	CA112f	Female	0.00000000	CA097m	Male	0.04761905
CA113f	Female	0.00000000	CA113f	Female	0.05462988	CA098m	Male	0.00000000
CA114f	Female	0.00000000	CA114f	Female	0.00000000	CA099m	Male	0.00000000
CA115f	Female	0.00000000	CA115f	Female	0.00000000	CA117m	Male	0.00000000
CA116f	Female	0.00000000	CA116f	Female	0.00000000	CA122m	Male	0.00000000
CA120f	Female	0.00000000	CA120f	Female	0.00000000			
CA121f	Female	0.00000000	CA121f	Female	0.00000000			
CA124f	Female	0.00000000	CA124f	Female	0.04179186			
CA125f	Female	0.00000000	CA125f	Female	0.00000000			
CA131f	Female	0.00000000	CA131f	Female	0.00000000			
CA133f	Female	0.00000000	CA133f	Female	0.00000000			
CA001m	Male	0.000000000	CA001m	Male	0.00000000			
CA002m	Male	0.000000000	CA002m	Male	0.00000000			
CA003m	Male	0.000000000	CA003m	Male	0.00000000			
CA004m	Male	0.000000000	CA004m	Male	0.00000000			
CA005m	Male	0.071111111	CA005m	Male	0.04490277			
CA006m	Male	0.045555556	CA006m	Male	0.00000000			
CA007m	Male	0.000000000	CA007m	Male	0.00000000			
CA009m	Male	0.000000000	CA009m	Male	0.00000000			
CA013m	Male	0.042222222	CA013m	Male	0.00000000			
CA018m	Male	0.000000000	CA018m	Male	0.00000000			
CA020m	Male	0.000000000	CA020m	Male	0.03606364			
CA021m	Male	0.025555556	CA021m	Male	0.00000000			
CA022m	Male	0.006666667	CA022m	Male	0.00000000			
CA023m	Male	0.045555556	CA023m	Male	0.02934590			
CA024m	Male	0.006666667	CA024m	Male	0.00000000			
CA025m	Male	0.000000000	CA025m	Male	0.00000000			
CA026m	Male	0.000000000	CA026m	Male	0.00000000			
CA027m	Male	0.061111111	CA027m	Male	0.04301709			
CA028m	Male	0.000000000	CA028m	Male	0.00000000			
CA031m	Male	0.000000000	CA031m	Male	0.00000000			
CA032m	Male	0.044444444	CA032m	Male	0.00000000			
CA033m	Male	0.000000000	CA033m	Male	0.00000000			
CA034m	Male	0.000000000	CA034m	Male	0.00000000			
CA035m	Male	0.000000000	CA035m	Male	0.00000000			
CA036m	Male	0.000000000	CA036m	Male	0.00000000			
CA037m	Male	0.000000000	CA037m	Male	0.00000000			
CA038m	Male	0.000000000	CA038m	Male	0.00000000			
CA040m	Male	0.000000000	CA040m	Male	0.04136712			
CA041m	Male	0.000000000	CA041m	Male	0.00000000			
CA043m	Male	0.000000000	CA043m	Male	0.05038303			
CA045m	Male	0.006666667	CA045m	Male	0.04725987			
CA048m	Male	0.000000000	CA048m	Male	0.00000000			
CA050m	Male	0.000000000	CA050m	Male	0.04655274			
CA052m	Male	0.000000000	CA052m	Male	0.00000000			
CA053m	Male	0.018888889	CA053m	Male	0.00000000			
CA054m	Male	0.000000000	CA054m	Male	0.00000000			
CA055m	Male	0.067777778	CA055m	Male	0.00000000			
CA057m	Male	0.061111111	CA057m	Male	0.00000000			
CA067m	Male	0.044444444	CA067m	Male	0.00000000			
CA068m	Male	0.030000000	CA068m	Male	0.04213318			
CA069m	Male	0.000000000	CA069m	Male	0.00000000			
CA072m	Male	0.000000000	CA072m	Male	0.00000000			
CA073m	Male	0.047777778	CA073m	Male	0.02934590			
CA074m	Male	0.006666667	CA074m	Male	0.03099588			
CA075m	Male	0.000000000	CA075m	Male	0.00000000			
CA076m	Male	0.030000000	CA076m	Male	0.04625810			
CA078m	Male	0.000000000	CA078m	Male	0.00000000			
CA079m	Male	0.000000000	CA079m	Male	0.02863877			
CA080m	Male	0.061111111	CA080m	Male	0.03170301			
CA082m	Male	0.030000000	CA082m	Male	0.00000000			
CA083m	Male	0.000000000	CA083m	Male	0.00000000			
CA084m	Male	0.030000000	CA084m	Male	0.03022982			
CA088m	Male	0.000000000	CA088m	Male	0.03606364			
CA091m	Male	0.000000000	CA091m	Male	0.00000000			
CA092m	Male	0.000000000	CA092m	Male	0.00000000			
CA096m	Male	0.039444444	CA096m	Male	0.00000000			
CA097m	Male	0.006666667	CA097m	Male	0.00000000			
CA098m	Male	0.006666667	CA098m	Male	0.00000000			
CA099m	Male	0.000000000	CA099m	Male	0.00000000			
CA103m	Male	0.070000000	CA103m	Male	0.00000000			
CA104m	Male	0.054444444	CA104m	Male	0.00000000			
CA105m	Male	0.000000000	CA105m	Male	0.00000000			
CA106m	Male	0.000000000	CA106m	Male	0.00000000			
CA107m	Male	0.000000000	CA107m	Male	0.00000000			
CA108m	Male	0.000000000	CA108m	Male	0.02333530			
CA109m	Male	0.039444444	CA109m	Male	0.00000000			
CA110m	Male	0.000000000	CA110m	Male	0.00000000			
CA111m	Male	0.000000000	CA111m	Male	0.00000000			
CA117m	Male	0.000000000	CA117m	Male	0.00000000			
CA118m	Male	0.000000000	CA118m	Male	0.00000000			
CA119m	Male	0.000000000	CA119m	Male	0.00000000			
CA122m	Male	0.000000000	CA122m	Male	0.00000000			
CA123m	Male	0.000000000	CA123m	Male	0.00000000			
CA126m	Male	0.000000000	CA126m	Male	0.00000000			
CA127m	Male	0.000000000	CA127m	Male	0.00000000			
CA128m	Male	0.000000000	CA128m	Male	0.00000000			
CA129m	Male	0.000000000	CA129m	Male	0.00000000			
CA130m	Male	0.000000000	CA130m	Male	0.00000000			
CA132m	Male	0.000000000	CA132m	Male	0.00000000			
CA134m	Male	0.000000000	CA134m	Male	0.00000000			
			UM1m	Male	0.00000000			
			UM10m	Male	0.04949912			
			UM11m	Male	0.00000000			
			UM12m	Male	0.00000000			
			UM13m	Male	0.03170301			
			UM14m	Male	0.04301709			
			UM15m	Male	0.04301709			
			UM2m	Male	0.05332940			
			UM3m	Male	0.00000000			
			UM4m	Male	0.00000000			
			UM5m	Male	0.04136712			
			UM6m	Male	0.00000000			
			UM7m	Male	0.00000000			
			UM8m	Male	0.04684738			
			UM9m	Male	0.05362404			

*Note*: See main text for additional details.

**TABLE A8:** Predictor variable measured for each *Crotalus atrox* subject of the present study.

ID	Sex	SVL	Body mass	Range length	MCP (ha)	Years monitored
CA100f	Female	835	560	1479.76	6.95	2
CA101f	Female	895	452	NA	NA	0
CA102f	Female	850	382	1742.58	10.01	3
CA010f	Female	850	346	NA	NA	0
CA112f	Female	795	363	NA	NA	0
CA113f	Female	912	538	NA	NA	0
CA114f	Female	640	255	NA	NA	0
CA115f	Female	620	177	NA	NA	0
CA116f	Female	650	180	NA	NA	0
CA011f	Female	860	370	NA	NA	0
CA120f	Female	690	240	714.34	3.08	2
CA121f	Female	655	300	701.94	2.83	2
CA124f	Female	880	541	925.4	5.03	1
CA125f	Female	770	394	NA	NA	0
CA012f	Female	950	430	NA	NA	0
CA131f	Female	865	546	NA	NA	0
CA133f	Female	835	593	NA	NA	0
CA014f	Female	815	404	1378.31	8.96	4
CA015f	Female	710	291	NA	NA	0
CA016f	Female	840	461	683.83	2.16	5
CA017f	Female	815	334	NA	NA	0
CA019f	Female	840	372	NA	NA	0
CA001f	Female	885	348	1511.80	7.77	7
CA029f	Female	850	413	1429.85	6.26	2
CA002f	Female	780	339	781.08	3.08	6
CA030f	Female	860	418	712.34	2.58	6
CA039f	Female	910	486	1929	13.91	2
CA003f	Female	870	395	NA	NA	0
CA042f	Female	845	359	611.94	2.34	3
CA044f	Female	830	534	907.04	3.29	4
CA046f	Female	890	307	798.02	2.36	5
CA047f	Female	830	398	1267.35	7.51	7
CA049f	Female	835	361	536.81	1.90	3
CA051f	Female	910	360	NA	NA	0
CA056f	Female	880	365	NA	NA	0
CA058f	Female	790	305	561.01	1.94	4
CA059f	Female	770	447	1354.99	8.08	2
CA060f	Female	825	354	NA	NA	0
CA061f	Female	840	503	2173.52	20.60	5
CA062f	Female	866	403	465.63	0.49945	1
CA063f	Female	910	386	NA	NA	0
CA064f	Female	940	513	407.71	0.93725	3
CA065f	Female	790	347	NA	NA	0
CA066f	Female	845	393	501.89	1.40	2
CA070f	Female	910	485	NA	NA	0
CA071f	Female	940	454	NA	NA	0
CA077f	Female	850	379	963.32	4.41	1
CA081f	Female	845	419.5	2085.79	7.23	3
CA085f	Female	775	243	NA	NA	0
CA086f	Female	350	18.5	NA	NA	0
CA087f	Female	705	257.3	NA	NA	0
CA089f	Female	310	21	NA	NA	0
CA008f	Female	845	347	NA	NA	0
CA090f	Female	298	19	NA	NA	0
CA093f	Female	825	399	944.4	3.35	3
CA094f	Female	775	370	633.28	1.87	2
CA095f	Female	905	476	648.33	1.82	1
CA103m	Male	910	505	NA	NA	0
CA104m	Male	900	429	NA	NA	0
CA105m	Male	1003	625	NA	NA	0
CA106m	Male	700	265	NA	NA	0
CA107m	Male	1040	905	NA	NA	0
CA108m	Male	810	405	NA	NA	0
CA109m	Male	1004	816	NA	NA	0
CA110m	Male	780	386	NA	NA	0
CA111m	Male	875	451	NA	NA	0
CA117m	Male	965	535	1326.72	6.02	2
CA118m	Male	875	453	NA	NA	0
CA119m	Male	1020	604	NA	NA	0
CA122m	Male	864	433	1351.75	9.18	1
CA123m	Male	786	384	NA	NA	0
CA126m	Male	900	632	NA	NA	0
CA127m	Male	935	600	NA	NA	0
CA128m	Male	1070	842	NA	NA	0
CA129m	Male	1060	775	NA	NA	0
CA130m	Male	1045	859	NA	NA	0
CA132m	Male	1015	740	NA	NA	0
CA134m	Male	738	243	NA	NA	0
CA013m	Male	1060	673	1723.93	17.85	3
CA018m	Male	950	470	NA	NA	0
CA001m	Male	NA	NA	NA	NA	0
CA020m	Male	850	452	NA	NA	0
CA021m	Male	960	563	NA	NA	0
CA022m	Male	880	NA	NA	NA	0
CA023m	Male	820	NA	NA	NA	0
CA024m	Male	950	642	NA	NA	0
CA025m	Male	800	333	NA	NA	0
CA026m	Male	840	456	NA	NA	0
CA027m	Male	1010	520	NA	NA	0
CA028m	Male	875	443	NA	NA	0
CA002m	Male	NA	NA	NA	NA	0
CA031m	Male	1000	472	3626.49	38.62	3
CA032m	Male	1100	745	1841.04	18.71	3
CA033m	Male	1105	694	1324.75	9.44	3
CA034m	Male	855	277	1404.21	6.09	2
CA035m	Male	810	404	NA	NA	0
CA036m	Male	1040	806	NA	NA	0
CA037m	Male	1095	665	1412.81	9.52	1
CA038m	Male	815	372	857.19	1.59	1
CA003m	Male	NA	NA	NA	NA	0
CA040m	Male	1030	726	NA	NA	0
CA041m	Male	935	537	1177.65	7.33	3
CA043m	Male	740	273	NA	NA	0
CA045m	Male	905	456	NA	NA	0
CA048m	Male	880	452	NA	NA	0
CA004m	Male	NA	NA	502.15	0.92945	1
CA050m	Male	940	531	2839.73	27.44	2
CA052m	Male	1040	746	NA	NA	0
CA053m	Male	850	493	NA	NA	0
CA054m	Male	860	368	NA	NA	0
CA055m	Male	760	339	2264.72	26.38	3
CA057m	Male	1140.5	973	NA	NA	0
CA005m	Male	820	374	1198.27	8.19	3
CA067m	Male	900	556	NA	NA	0
CA068m	Male	1050	736	NA	NA	0
CA069m	Male	915	631	NA	NA	0
CA006m	Male	795	342	1592.53	14.05	4
CA072m	Male	850	512	NA	NA	0
CA073m	Male	695	284	NA	NA	0
CA074m	Male	920	582	NA	NA	0
CA075m	Male	721	295	NA	NA	0
CA076m	Male	958	656	1239.26	5.83	2
CA078m	Male	1015	831	NA	NA	0
CA079m	Male	925	650	1337.83	11.46	2
CA007m	Male	870	456	1163.76	5.18	2
CA080m	Male	945	547	NA	NA	0
CA082m	Male	875	597.5	NA	NA	0
CA083m	Male	850	413.5	NA	NA	0
CA084m	Male	980	633	NA	NA	0
CA088m	Male	1005	622	NA	NA	0
CA091m	Male	320	24	NA	NA	0
CA092m	Male	980	686	1491.76	13.33	1
CA096m	Male	920	570	2366.75	21.94	2
CA097m	Male	965	653	2024.56	16.69	2
CA098m	Male	1040	859	2334.71	17.95	2
CA099m	Male	1065	735	NA	NA	0
CA009m	Male	990	517	NA	NA	0

Abbreviations: MCP, home range size minimum convex polygon; SVL, snout–vent length.
