# Supplementary material for: Fission–fusion dynamics in the social networks of a North American pitviper
Source: Ecol Evol. 2023 Aug 7;13(8):e10339. doi: 10.1002/ece3.10339 (PMC10405236; doi:10.1002/ece3.10339)
Supplement: Supplementary file 1 — Appendix S1 [file ECE3-13-e10339-s001.docx]

**Supplementary Material from “Fission-Fusion Dynamics in the Social Networks of a North American Pitviper”**

**Rattlesnakes in social network analysis**

Numerous moderate- to large-sized species of rattlesnakes (e.g., *Crotalus atrox, C. cerberus, C*. *oreganus*, *C. stephensi*, and *C. viridis*) possess several attributes that allow their study in nature to be manageable and thus good candidate models for longitudinal studies using network analysis. The changing academic milieu and publication stance on social behavior in animals, in general, and rattlesnakes and other reptiles, in particular, is summarized by Schuett and colleagues [1, 2] and inspired by Doody and colleagues [3, 4] and Van Dyke and colleagues [5].

1) In the five rattlesnake taxa mentioned above, individuals often assemble to form highly localized groups (two to several hundred) in communal winter shelters (communal dens) from fall to spring, or even longer [1, 2, 6–9]. Rarely observed in other species of snakes, this attribute permits one not only to observe most adult snakes (and sometimes neonates and juveniles) of a population [1, 2, 8, 10–14], but also to capture them for processing such as inserting permanent PIT tags (identification), performing radio-telemetry surgery (radio-tracking), and procuring tissues (e.g., blood, scale clips) for subsequent DNA analysis [2, 9]. See Schuett and colleagues [1, 2, 11] for a discussion of other attributes of communal shelters in rattlesnakes for studies of social behavior.

2) With the advent of affordable commercial radio-telemetry for terrestrial snakes since the late 1980s, it is now possible to implant radio-transmitters into the body cavity of moderate- to large-sized individuals for long periods (e.g., months to years) before removal or replacement [15]. Radio-telemetry provides a unique radio-signal for each individual. Coupled with the use of PIT tags and other forms of identification methods (e.g., unique painting of rattles, photographs), radio-tracking and locating individuals can be done with high precision.

3) As a group, rattlesnakes tend to be slow-moving, ambush (sit-and-wait) predators [16, 17]. Consequently, their movements are limited and distances traveled per movement session (e.g., evening) tend to be short (e.g. 0.1–1km). Thus, unlike many other organisms such as birds, felids, and ungulates, radio-tracking can be done by foot and managed on a daily basis if needed. Furthermore, when compared to other snake species, rattlesnakes as sit-and-wait predators are often exposed on the ground-surface and visible for observation purposes [17; R. Repp and G. Schuett, pers. observ., 2001–2015].

4) Nearly all large species of rattlesnakes are long-lived, with some attaining lifespans exceeding four decades [18–19]. Longevity is especially desirable in that overlapping generations can be studied simultaneously and by a single researcher. Maturation in females is from 3 to 12 years dependent on the species and location [18–19].

**Communal denning and relatedness analysis**

The hypothesis of communal denning and relatedness was investigated earlier [2] with a larger number of adult *C. atrox* (Suizo Mountains) than the focal group used in the current study. The focal group in the current study all were fitted with radio-transmitters (N = 50; 22 males, 28 females). The earlier study [2] incorporated animals without radio-transmitters.

A grand total of 191 adult *C. atrox* were genotyped [9; see 2, pp. 196-198]. Of these 191 subjects, 46 (22 ♂, 28 ♀) were fitted radio-transmitters and 50 were known to occupy one of seven different communal dens [2]. We used the maximum likelihood method implemented in ML-relate [20]. See Jones and colleagues [21] on methods to calculate pairwise relatedness (R) and estimate the relationship of all pairs [2, 9]. We ascertained relationships among the subjects that shared communal dens, and used bootstrapping to compare mean relatedness between pairs of individuals that shared communal dens with random pairs drawn from the population as a whole.

An initial test pooling all den occupants indicated that individuals that shared dens, although often unrelated, had a higher average relatedness than random pairs (mean R = 0.029, *p* < 0.001). When individual dens were analyzed separately, however, four of the seven dens in the sample had pairs of subjects that were identified as at least half-sibs, but three dens had no detectable relatives (Supporting Information, Tables S1–S8).

Accordingly, this analysis supports the view that kin-relationships might be drivers, in part, of the dynamics of communal denning in *C. atrox* at this site. Furthermore, even in this analysis, we suspect under-sampling is still at play owing to the difficulty in capturing all of the den occupants [GW Schuett, pers. observ., 2001–2015]. Accordingly, kin-relations may be yet further underestimated, especially in the three dens where no relatives were detected.

**Table S1.** Genotype results of adult Western Diamond-backed Rattlesnakes (*Crotalus atrox*) from Den AD-1 at the Suizo Mountains, AZ, USA. Site was sampled from 2001 to 2010. HS = half-sibs [2, 9].

| Matrix of Relatedness | |  | CAMD2_S1b | CASD-002F | CAMD2_S10 | CAMD2_S7 | CAMD2_S5 | CAMD2_S6 | CAMD2_S2b | CAMD2_S4b | CASD-033 | CASD-038 | CASD-080 | CASD-083 |
| --- | --- | --- | --- | --- | --- | --- | --- | --- | --- | --- | --- | --- | --- | --- |
|  |  |  | F | F | M | M | M | M | M | M | M | M | M | M |
|  |  |  | CA-1 | CA-2 | CA-1 | CA-2 | CA-3 | CA-4 | CA-5 | CA-6 | CA-33 | CA-38 | CA-80 | CA-83 |
| CAMD2_S1b | F | CA-1 | x |  |  |  |  |  |  |  |  |  |  |  |
| CASD-002F | F | CA-2 | 0 | x |  |  |  |  |  |  |  |  |  |  |
| CAMD2_S10 | M | CA-1 | 0.03 | 0 | x |  |  |  |  |  |  |  |  |  |
| CAMD2_S7 | M | CA-2 | 0 | 0 | 0.06 | x |  |  |  |  |  |  |  |  |
| CAMD2_S5 | M | CA-3 | 0.03 | 0 | 0.17 | 0.06 | x |  |  |  |  |  |  |  |
| CAMD2_S6 | M | CA-4 | 0 | 0 | 0.04 | 0 | 0.01 | x |  |  |  |  |  |  |
| CAMD2_S2b | M | CA-5 | 0.03 | 0.03 | 0 | 0.08 | 0.03 | 0.06 | x |  |  |  |  |  |
| CAMD2_S4b | M | CA-6 | 0 | 0.03 | 0 | 0 | 0 | 0.1 | 0 | x |  |  |  |  |
| CASD-033 | M | CA-33 | 0.01 | 0.13 | 0 | 0 | 0 | 0.12 | 0 | 0.03 | x |  |  |  |
| CASD-038 | M | CA-38 | 0 | 0 | 0 | 0 | 0.01 | 0.03 | 0 | 0.09 | 0.09 | x |  |  |
| CASD-080 | M | CA-80 | 0.04 | 0 | 0 | 0 | 0 | 0 | 0 | 0 | 0.03 | 0 | x |  |
| CASD-083 | M | CA-83 | 0 | 0 | 0.03 | 0 | 0.07 | 0 | 0 | 0.06 | 0 | 0.39 | 0 | x |
|  |  |  |  |  |  |  |  |  |  |  |  |  |  |  |
| Matrix of Relationship | |  | CAMD2_S1b | CASD-002F | CAMD2_S10 | CAMD2_S7 | CAMD2_S5 | CAMD2_S6 | CAMD2_S2b | CAMD2_S4b | CASD-033 | CASD-038 | CASD-080 | CASD-083 |
|  |  |  | F | F | M | M | M | M | M | M | M | M | M | M |
|  |  |  | CA-1 | CA-2 | CA-1 | CA-2 | CA-3 | CA-4 | CA-5 | CA-6 | CA-33 | CA-38 | CA-80 | CA-83 |
| CAMD2_S1b | F | CA-1 | x |  |  |  |  |  |  |  |  |  |  |  |
| CASD-002F | F | CA-2 | u | x |  |  |  |  |  |  |  |  |  |  |
| CAMD2_S10 | M | CA-1 | u | u | x |  |  |  |  |  |  |  |  |  |
| CAMD2_S7 | M | CA-2 | u | u | u | x |  |  |  |  |  |  |  |  |
| CAMD2_S5 | M | CA-3 | u | u | HS | u | x |  |  |  |  |  |  |  |
| CAMD2_S6 | M | CA-4 | u | u | u | u | u | x |  |  |  |  |  |  |
| CAMD2_S2b | M | CA-5 | u | u | u | u | u | u | x |  |  |  |  |  |
| CAMD2_S4b | M | CA-6 | u | u | u | u | u | u | u | x |  |  |  |  |
| CASD-033 | M | CA-33 | u | HS | u | u | u | u | u | u | x |  |  |  |
| CASD-038 | M | CA-38 | u | u | u | u | u | u | u | u | u | x |  |  |
| CASD-080 | M | CA-80 | u | u | u | u | u | u | u | u | u | u | x |  |
| CASD-083 | M | CA-83 | u | u | u | u | u | u | u | u | u | HS | u | x |

**Table S2.** Genotype results of adult Western Diamond-backed Rattlesnakes (*Crotalus atrox*) from Den AD-4 at the Suizo Mountains, AZ, USA. Site was sampled from 2001 to 2010. HS = half-sibs. See Clark et al. (2014) and Schuett et al. (2014).

| Matrix of relatedness | |  | CASD-016 | CASD-081 | CAMD2_S16 | CASD-032 | CAMD2_S25 | CASD-050 | CASD-055 | CASD-073 | CASD-074 | CASD-110 | CASD-x053 |
| --- | --- | --- | --- | --- | --- | --- | --- | --- | --- | --- | --- | --- | --- |
|  |  |  | F | F | M | M | M | M | M | M | M | M | M |
|  |  |  | CA-16 | CA-81 | CA-13 | CA-32 | CA-37 | CA-50 | CA-55 | CA-73 | CA-74 | CA-110 | CA-X53 |
| CASD-016 | F | CA-16 | x |  |  |  |  |  |  |  |  |  |  |
| CASD-081 | F | CA-81 | 0.015 | x |  |  |  |  |  |  |  |  |  |
| CAMD2_S16 | M | CA-13 | 0 | 0.01 | x |  |  |  |  |  |  |  |  |
| CASD-032 | M | CA-32 | 0 | 0 | 0.058 | x |  |  |  |  |  |  |  |
| CAMD2_S25 | M | CA-37 | 0 | 0 | 0.06 | 0.13 | x |  |  |  |  |  |  |
| CASD-050 | M | CA-50 | 0 | 0 | 0.03 | 0.1 | 0.02 | x |  |  |  |  |  |
| CASD-055 | M | CA-55 | 0 | 0 | 0 | 0 | 0.02 | 0.13 | x |  |  |  |  |
| CASD-073 | M | CA-73 | 0.12 | 0.05 | 0 | 0.21 | 0 | 0.38 | 0 | x |  |  |  |
| CASD-074 | M | CA-74 | 0 | 0 | 0.03 | 0 | 0.14 | 0 | 0.09 | 0 | x |  |  |
| CASD-110 | M | CA-110 | 0.06 | 0.08 | 0 | 0 | 0 | 0 | 0.03 | 0 | 0.05 | x |  |
| CASD-x053 | M | CA-X53 | 0 | 0.04 | 0.1148 | 0.0603 | 0 | 0.1215 | 0.1334 | 0.1405 | 0 | 0 | x |
|  |  |  |  |  |  |  |  |  |  |  |  |  |  |
| Matrix of relationship | |  | CASD-016 | CASD-081 | CAMD2_S16 | CASD-032 | CAMD2_S25 | CASD-050 | CASD-055 | CASD-073 | CASD-074 | CASD-110 | CASD-x053 |
|  |  |  | F | F | M | M | M | M | M | M | M | M | M |
|  |  |  | CA-16 | CA-81 | CA-13 | CA-32 | CA-37 | CA-50 | CA-55 | CA-73 | CA-74 | CA-110 | CA-X53 |
| CASD-016 | F | CA-16 | x |  |  |  |  |  |  |  |  |  |  |
| CASD-081 | F | CA-81 | u | x |  |  |  |  |  |  |  |  |  |
| CAMD2_S16 | M | CA-13 | u | u | x |  |  |  |  |  |  |  |  |
| CASD-032 | M | CA-32 | u | u | u | x |  |  |  |  |  |  |  |
| CAMD2_S25 | M | CA-37 | u | u | u | HS | x |  |  |  |  |  |  |
| CASD-050 | M | CA-50 | u | u | u | u | u | x |  |  |  |  |  |
| CASD-055 | M | CA-55 | u | u | u | u | u | u | x |  |  |  |  |
| CASD-073 | M | CA-73 | HS | u | u | HS | u | HS | u | x |  |  |  |
| CASD-074 | M | CA-74 | u | u | u | u | HS | u | u | u | x |  |  |
| CASD-110 | M | CA-110 | u | u | u | u | u | u | u | u | u | x |  |
| CASD-x053 | M | CA-X53 | u | u | HS | u | u | HS | HS | HS | 0 | 0 | x |

**Table S3.** Genotype results of adult Western Diamond-backed Rattlesnakes (*Crotalus atrox*) from Den AD-5 at the Suizo Mountains, AZ, USA. Site was sampled from 2001 to 2010. HS = half-sibs. See Clark et al. (2014) and Schuett et al. (2014).

| Relatedness matrix | |  | CASD-077 | CASD-034 | CASD-043 | CAMD2_S25 | CASD-045 |
| --- | --- | --- | --- | --- | --- | --- | --- |
|  |  |  | F | M | M | M | M |
|  |  |  | CA-77 | CA-34 | CA-43 | CA-37 | CA-45 |
| CASD-077 | F | CA-77 | x |  |  |  |  |
| CASD-034 | M | CA-34 | 0 | x |  |  |  |
| CASD-043 | M | CA-43 | 0.08 | 0 | x |  |  |
| CAMD2_S25 | M | CA-37 | 0.1029 | 0.0444 | 0.1027 | x |  |
| CASD-045 | M | CA-45 | 0.06 | 0 | 0.004 | 0 | x |
|  |  |  |  |  |  |  |  |
| Relatinoship matrix | |  | CASD-077 | CASD-034 | CASD-043 | CAMD2_S25 | CASD-045 |
|  |  |  | F | M | M | M | M |
|  |  |  | CA-77 | CA-34 | CA-43 | CA-37 | CA-45 |
| CASD-077 | F | CA-77 | x |  |  |  |  |
| CASD-034 | M | CA-34 | u | x |  |  |  |
| CASD-043 | M | CA-43 | u | u | x |  |  |
| CAMD2_S25 | M | CA-37 | HS | u | HS | x |  |
| CASD-045 | M | CA-45 | u | u | u | u | x |
|  |  |  |  |  |  |  |  |

**Table S4.** Genotype results of adult Western Diamond-backed Rattlesnakes (*Crotalus atrox*) from Den AD-6 at the Suizo Mountains, AZ, USA. Site was sampled from 2001 to 2010. HS = half-sibs. See Clark et al. (2014) and Schuett et al. (2014).

| Relatedness matrix |  |  | CASD-044 | CASD-046 | CAMD007 | CASD-068 | CASD-082 | CASD-084 | CASD-111 |
| --- | --- | --- | --- | --- | --- | --- | --- | --- | --- |
|  |  |  | F | F | M | M | M | M | M |
|  |  |  | CA-44 | CA-46 | CA-7 | CA-68 | CA-82 | CA-84 | CA-111 |
| CASD-044 | F | CA-44 | x |  |  |  |  |  |  |
| CASD-046 | F | CA-46 | 0.04 | x |  |  |  |  |  |
| CAMD007 | M | CA-7 | 0 | 0.03 | x |  |  |  |  |
| CASD-068 | M | CA-68 | 0.03 | 0.05 | 0 | x |  |  |  |
| CASD-082 | M | CA-82 | 0.06 | 0 | 0 | 0.03 | x |  |  |
| CASD-084 | M | CA-84 | 0.04 | 0.05 | 0 | 0.03 | 0.05 | x |  |
| CASD-111 | M | CA-111 | 0.04 | 0 | 0 | 0.04 | 0.03 | 0 | x |
|  |  |  |  |  |  |  |  |  |  |
| Relationship matrix | |  | CASD-044 | CASD-046 | CAMD007 | CASD-068 | CASD-082 | CASD-084 | CASD-111 |
|  |  |  | F | F | M | M | M | M | M |
|  |  |  | CA-44 | CA-46 | CA-7 | CA-68 | CA-82 | CA-84 | CA-111 |
| CASD-044 | F | CA-44 | x |  |  |  |  |  |  |
| CASD-046 | F | CA-46 | u | x |  |  |  |  |  |
| CAMD007 | M | CA-7 | u | u | x |  |  |  |  |
| CASD-068 | M | CA-68 | u | u | u | x |  |  |  |
| CASD-082 | M | CA-82 | u | u | u | u | x |  |  |
| CASD-084 | M | CA-84 | u | u | u | u | u | x |  |
| CASD-111 | M | CA-111 | u | u | u | u | u | u | x |

**Table S5.** Genotype results of adult Western Diamond-backed Rattlesnakes (*Crotalus atrox*) from Den AD-7 at the Suizo Mountains, AZ, USA. Site was sampled from 2001 to 2010. HS = half-sibs. See Clark et al. (2014) and Schuett et al. (2014).

| Relatedness matrix |  |  | CASD-047 | CASD-058 | CASD-040 | CASD-076 | CASD-078 | CASD-079 | CASD-092 | CASD-043 | CASD-073 | CASD-074 | CASD-X057 |
| --- | --- | --- | --- | --- | --- | --- | --- | --- | --- | --- | --- | --- | --- |
|  |  |  | F | F | M | M | M | M | M | M | M | M | M |
|  |  |  | CA-47 | CA-58 | CA-40 | CA-76 | CA-78 | CA-79 | CA-92 | CA-43 | CA-73 | CA-74 | CA-x57 |
| CASD-047 | F | CA-47 | x |  |  |  |  |  |  |  |  |  |  |
| CASD-058 | F | CA-58 | 0 | x |  |  |  |  |  |  |  |  |  |
| CASD-040 | M | CA-40 | 0 | 0 | x |  |  |  |  |  |  |  |  |
| CASD-076 | M | CA-76 | 0 | 0.02 | 0.06 | x |  |  |  |  |  |  |  |
| CASD-078 | M | CA-78 | 0 | 0 | 0.09 | 0 | x |  |  |  |  |  |  |
| CASD-079 | M | CA-79 | 0 | 0.06 | 0.06 | 0.03 | 0 | x |  |  |  |  |  |
| CASD-092 | M | CA-92 | 0.02 | 0 | 0 | 0 | 0 | 0 | x |  |  |  |  |
| CASD-043 | M | CA-43 | 0 | 0 | 0.02 | 0 | 0 | 0.03 | 0 | x |  |  |  |
| CASD-073 | M | CA-73 | 0.002 | 0.01 | 0 | 0.06 | 0 | 0 | 0 | 0 | x |  |  |
| CASD-074 | M | CA-74 | 0 | 0.007 | 0.06 | 0.07 | 0 | 0 | 0 | 0 | 0 | x |  |
| CASD-X057 | M | CA-x57 | 0 | 0.02 | 0 | 0 | 0.08 | 0 | 0 | 0.003 | 0 | 0.05 | x |
|  |  |  |  |  |  |  |  |  |  |  |  |  |  |
| Relationship matrix | |  | CASD-047 | CASD-058 | CASD-040 | CASD-076 | CASD-078 | CASD-079 | CASD-092 | CASD-043 | CASD-073 | CASD-074 | CASD-X057 |
|  |  |  | F | F | M | M | M | M | M | M | M | M | M |
|  |  |  | CA-47 | CA-58 | CA-40 | CA-76 | CA-78 | CA-79 | CA-92 | CA-43 | CA-73 | CA-74 | CA-x57 |
| CASD-047 | F | CA-47 | x |  |  |  |  |  |  |  |  |  |  |
| CASD-058 | F | CA-58 | u | x |  |  |  |  |  |  |  |  |  |
| CASD-040 | M | CA-40 | u | u | x |  |  |  |  |  |  |  |  |
| CASD-076 | M | CA-76 | u | u | u | x |  |  |  |  |  |  |  |
| CASD-078 | M | CA-78 | u | u | u | u | x |  |  |  |  |  |  |
| CASD-079 | M | CA-79 | u | u | u | u | u | x |  |  |  |  |  |
| CASD-092 | M | CA-92 | u | u | u | u | u | u | x |  |  |  |  |
| CASD-043 | M | CA-43 | u | u | u | u | u | u | u | x |  |  |  |
| CASD-073 | M | CA-73 | u | u | u | u | u | u | u | u | x |  |  |
| CASD-074 | M | CA-74 | u | u | u | u | u | u | u | u | u | x |  |
| CASD-X057 | M | CA-x57 | u | u | u | u | u | u | u | u | u | u | x |

**Table S6.** Genotype results of adult Western Diamond-backed Rattlesnakes (*Crotalus atrox*) from Den AD-8 at the Suizo Mountains, AZ, USA. Site was sampled from 2001 to 2010. HS = half-sibs. See Clark et al. (2014) and Schuett et al. (2014).

| Relatedness matrix |  |  | CASD-064 | CASD-101 | CASD-102 | CASD-097 |
| --- | --- | --- | --- | --- | --- | --- |
|  |  |  | F | F | F | M |
|  |  |  | CA-64 | CA-101 | CA-102 | CA-97 |
| CASD-064 | F | CA-64 | x |  |  |  |
| CASD-101 | F | CA-101 | 0 | x |  |  |
| CASD-102 | F | CA-102 | 0.07 | 0.04 | x |  |
| CASD-097 | M | CA-97 | 0 | 0.01 | 0 | x |
|  |  |  |  |  |  |  |
| Relationship matrix | |  | CASD-064 | CASD-101 | CASD-102 | CASD-097 |
|  |  |  | F | F | F | M |
|  |  |  | CA-64 | CA-101 | CA-102 | CA-97 |
| CASD-064 | F | CA-64 | x |  |  |  |
| CASD-101 | F | CA-101 | u | x |  |  |
| CASD-102 | F | CA-102 | u | u | x |  |
| CASD-097 | M | CA-97 | u | u | u | x |

**Table S7.** Genotype results of adult Western Diamond-backed Rattlesnakes (*Crotalus atrox*) from Den-9 at the Suizo Mountains, AZ, USA. Site was sampled from 2001 to 2010. HS = half-sibs. See Clark et al. (2014) and Schuett et al. (2014).

| R matrix |  |  | CASD-040 | CASD-052 | CASD-053 |
| --- | --- | --- | --- | --- | --- |
|  |  |  | M | M | M |
|  |  |  | CA-40 | CA-52 | CA-53 |
| CASD-040 | M | CA-40 | x |  |  |
| CASD-052 | M | CA-52 | 0 | x |  |
| CASD-053 | M | CA-53 | 0.24 | 0.02 | x |
|  |  |  |  |  |  |
| Relationship matrix | |  | CASD-040 | CASD-052 | CASD-053 |
|  |  |  | M | M | M |
|  |  |  | CA-40 | CA-52 | CA-53 |
| CASD-040 | M | CA-40 | x |  |  |
| CASD-052 | M | CA-52 | U | x |  |
| CASD-053 | M | CA-53 | HS | U | x |

**Table S8.** Genotype results of adult Western Diamond-backed Rattlesnakes (*Crotalus atrox*) from Den 1 at the Suizo Mountains, AZ, USA. Site was sampled from 2001 to 2010. HS = half-sibs. See Clark et al. (2014) and Schuett et al. (2014). There was no relatedness between these two male snakes.

| CASD-096 | M | CA-96 |
| --- | --- | --- |
| CASD-x023 | M | CA-x23 |

**References**

1. Schuett GW, Repp RA, Spencer CL, Beamann K, Painter CW. 2016a *Crotalus atrox* (western

diamond-backed rattlesnake). In *Rattlesnakes of Arizona*, *vol. 1* (eds. GW Schuett, MJ Feldner, CF

Smith, RS Reiserer), pp. 333–394. Rodeo, NM: ECO Publishing.

2. Schuett GW, Clark RW, Repp RA, Amarello M, Smith CF, Greene HW. 2016b Social behavior of

rattlesnakes: a shifting paradigm. In *Rattlesnakes of Arizona*, *vol. 2* (eds. GW Schuett, MJ Feldner, CF

Smith, RS Reiserer), pp. 161–244. Rodeo, NM: ECO Publishing.

3. Doody JS, Burghardt GM, Dinets V, Hauber M. 2013 Breaking the social-non-social dichotomy: a role

for reptiles in vertebrate social behavior research? *Ethology* **119**, 95–103.

4. Doody SJ, Dinets V, Burghardt GM. 2021 *The secret social lives of reptiles*. Baltimore, MD: Johns

Hopkins University Press.

5. Van Dyke JU, Thompson MB, Burridge CP, Castelli MA, Clulow S, Dissanayake, DSB, Dong CM,

Doody JS, Edwards DL, Ezaz, T, Friesen CR, Gardner MG, et al. 2021 Australian lizards are

outstanding models for reproductive biology research. *Aust. J. Zool*. **68**, 168–199.

6. Klauber LM. 1956 *Rattlesnakes. Their habits, life histories, and influence on mankind*, *2 vols.* Berkeley

and Los Angeles, CA: University of California Press.

7. Sexton O, Jacobson JP, Bramble JE. 1992 Geographic variation in some activities associated with

hibernation in Neartic pitvipers. In *Biology of the pitvipers* (eds. JA Campbell, ED Brodie, Jr.), pp.

337–346. Tyler, TX: Selva.

8. Amarello M. 2012 Social Snakes? Non-random association patterns detected in a population of Arizona

black rattlesnakes (*Crotalus cerberus*). Unpublished thesis. Arizona State University, Tempe, AZ:

USA.

9. Clark RW, Schuett GW, Repp RA, Amarello M, Smith CF, Herrmann H-W. 2014 Mating systems,

reproductive success, and sexual selection in a secretive species: a case study of the western diamond-

backed rattlesnake, *Crotalus atrox*. *PLoS ONE* **9**, e90616.

10. Beck DD. 1995 Ecology and energetics of three sympatric rattlesnake species in the Sonoran Desert.

*J. Herpetol*. **29**, 211–223.

11. Repp RA. 1998 Wintertime observations on five species of reptiles in the Tucson area:

shelter site selections/fidelity to shelter sites/notes on behavior. *Bull. Chicago Herpetol. Soc.*

**33**, 49–56.

12. Diller LV, Wallace RL. 2002 Growth, reproduction and survival in a population of *Crotalus viridis* in

north central Idaho. *Herpetol. Monogr*. **16**, 26–45.

13. Ashton KG. 2003 Movements and mating behavior of adult male midget faded rattlesnakes, *Crotalus*

*oreganus concolor*, in Wyoming. *Copeia* **2003**, 190–194.

14. Repp RA, Schuett GW 2008 Western diamond-backed rattlesnakes, *Crotalus*

*atrox* (Serpentes: Viperidae), gain water by harvesting and drinking rain, sleet, and

snow. *Southwest. Nat.* **53**, 108–114.

15. Beaupre SJ 2016 Novel tracking and remote monitoring technologies: Applications to

studying wild rattlesnakes. In *Rattlesnakes of Arizona*, vol. 2 (eds. GW Schuett, MJ Feldner,

CF Smith, RS Reiserer), pp. 58–91. Rodeo, NM: ECO Publishing

16. Nowak EN, Theimer TC, Schuett GW. 2008 Functional and numeric responses of predators: where do

vipers fit in the traditional paradigms? *Biol. Rev*. **83**, 601–620.

17. Clark RW. 2016 The hunting and feeding behavior of wild rattlesnakes. In *Rattlesnakes of Arizona*,

*vol. 2* (eds. GW Schuett, MJ Feldner, CF Smith, RS Reiserer), pp. 91–118. Rodeo, NM: ECO

Publishing.

18. Brown WS. 1993 Biology, status, and management of the timber rattlesnake (*Crotalus horridus*): a

guide for conservation. *Herpetol. Circ.* **22**, 1–78.

19. Brown WS. 2016 Lifetime reproduction in a northern metapopulation of timber rattlesnakes (*Crotalus*

*horridus*). *Herpetologica* **72**, 331– 342.

20. Kalinowski ST, Taper ML (2006) Maximum likelihood estimation of the frequency of null alleles at

microsatellite loci. *Conserv. Genet*. **7**, 991–995

21. Jones A, Clayton M, Small K, Paczolt A, Ratterman N. 2010 A practical guide to methods of

parentage analysis. *Mol. Ecol. Res*. **10**, 6–30.
